# Discovery of Levesquamide B through Global Natural Product Social Molecular Networking

**DOI:** 10.3390/molecules27227794

**Published:** 2022-11-12

**Authors:** Mary M. LeClair, Zacharie A. Maw, Alyssa L. Grunwald, Joshua R. Kelly, Bradley A. Haltli, Russell G. Kerr, Christopher Cartmell

**Affiliations:** 1 Department of Chemistry, University of Prince Edward Island, 550 University Avenue, Charlottetown, PE C1A 4P3, Canada; 2 Department of Biomedical Sciences, Atlantic Veterinary College, 550 University Avenue, Charlottetown, PE C1A 4P3, Canada; 3 Nautilus Biosciences Croda, 550 University Avenue, Charlottetown, PE C1A 4P3, Canada; 4 Antimicrobial Discovery Center, Department of Biology, Northeastern University, Boston, MA 02115, USA

**Keywords:** levesquamide, GNPS, marine natural product, *Streptomyces*

## Abstract

Levesquamide A is an isothiazolinone-containing anti-tubercular natural product isolated from *Streptomyces* sp. RKND-216. Through the use of Global Natural Product Social Molecular Networking (GNPS), additional members of the levesquamide family were identified (B-G). Levesquamide B is a glycosylated analogue, isolated and structurally elucidated via spectroscopical techniques along with the putative structures of levesquamide C and D. For masses relating to the additional three levesquamides (E-G), their complete structures remain ambiguous.

## 1. Introduction

The medicinal importance of natural products cannot be overlooked with over 70% of antimicrobials and 60% of anticancer agents over the past 4 decades being derived from natural products in one form or another [1]. *Actinobacteria* are one of nature’s most prolific medicinal chemists producing a plethora of antibiotic, anticancer, antifungal and anti-infective agents [2,3,4]. Historically, *Actinobacteria* were isolated from terrestrial habitats; however, to increase the probability of discovering novel bioactive natural products, researchers have turned to more unique habitats [5]. Marine environments have proven to be a rich source for bioprospecting for new natural products due to high levels of biodiversity and the prevalence of symbiotic relationships between microorganisms and sessile invertebrates [6]. The use of marine microorganisms as a source of therapeutic agents is a relatively recent approach when compared to the investigation of their terrestrial relatives. [7] With only a small fraction of marine microbial diversity explored to date, this resource represents a huge treasure trove of untapped potential for the discovery of much needed new therapeutic agents [8].

**Figure 1:**
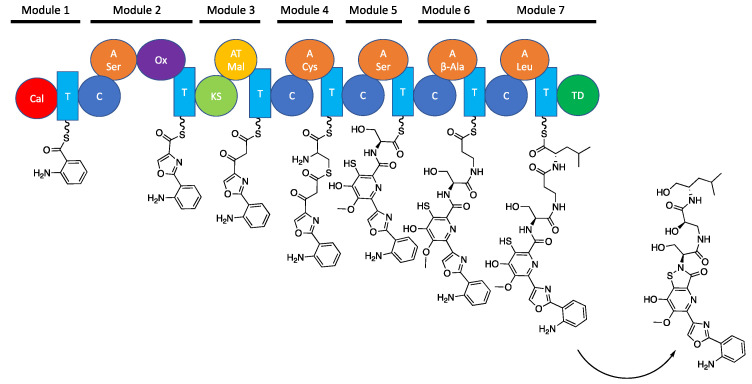
Proposed biosynthetic pathway for the levesquamides.

As part of a continuing effort to discover new antimicrobial agents from marine bacteria we recently described the discovery of levesquamide, an isothiazolinone-containing antitubercular natural product, from *Streptomyces* sp. RKND-216 which was isolated from marine sediment collected in Prince Edward Island, Canada [9]. The levesquamide biosynthetic gene cluster (BGC) was identified in the *Streptomyces* sp. RKND-216 genome and its involvement in levesquamide biosynthesis established through gene inactivation and complementation experiments [9,10]. Biosynthesis of levesquamide is achieved via a non-colinear mixed non-ribosomal peptide synthetase (NRPS)—polyketide synthase (PKS) thiotemplate-directed mechanism mediated by LvqA-D, which collectively encode seven modules. Each module catalyzes the incorporation of an amino acid or two carbon polyketide extender (module 3) unit into the structure of levesquamide according to established PKS and NRPS biosynthetic mechanisms [11]. Levesquamide biosynthesis starts with the activation of anthranilate by module 1. Module 2 activates L-serine and catalyzes its condensation with anthranilate and the subsequent cyclization and oxidation reactions required to form a five-membered oxazole ring using the hydroxyl side chain of the serine residue. Next, the PKS-encoding module 3 activates malonyl-CoA and incorporates an acetate into the levesquamide structure. Module 4 is predicted to incorporate L-cysteine by catalyzing chain elongation between the sulfhydryl of the cysteine and the carbonyl of the preceding acetate. The condensation (C) domain of module 4 is a cyclization-type C domain and is proposed to form a 7-membered heterocycle that undergoes a rearrangement to release the cysteine thiol to form a six membered ring [10]. This proposal was based on homology of modules 3 and 4 encoded on LvqB to the ClmN1 and CaeA2 enzymes responsible for forming the second pyridine ring it the 2,2′-bipyridine ring system in collismycin and caerulomycin biosynthesis [12,13] The resulting 6-membered heterocycle would then undergo dehydration, oxidation and methylation to complete the decoration of the pyridine ring. NRPS modules 5 to 7 sequentially incorporate L-serine, β-alanine and L-leucine, respectively. Module 7 contains a terminal reductase domain that is predicted to release the levesquamide intermediate as an alcohol, which is consistent with the presence of a leucinol terminal residue in the levesquamide. Following release from the NRPS the alpha carbon of the β-alanine residue likely undergoes hydroxylation to form D-isoserine and the isothiazolinone moiety is formed via condensation between the C3 thiol and the amine of the serine incorporated by module 5 by a speculated oxidative pathway involving a sulfenic acid intermediate [10] (Figure 1).

Molecular networking (GNPS) has become a pivotal tool in the deconvolution of mass spectrometry data sets whilst also combatting the ever-present issue of rediscovery of known natural products. Since its introduction in 2012 this chemi-informatic tool’s popularity has soared, enabling visualization and annotation of aligned MS^2^ spectra connecting related molecules via spectral similarity scores [14,15].

**Figure 2:**
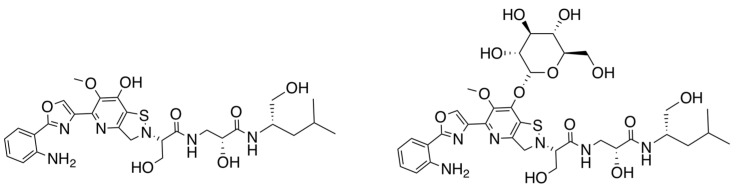
Structures of levesquamide A (**left**) and levesquamide B (**right**).

Intrigued by the biosynthetic potential of *Streptomyces* sp. RKND-216 and the structural novelty of levesquamide, reinvestigation led to the identification of 7 levesquamide analog. We describe here the isolation, characterization and structural elucidation of a glycosylated analogue, levesquamide B and putative structures of levesquamides C and D (See Supplementary, Figures S9 and S10). We also here provide masses and fragmentation of a further three levesquamide analogues (E-G), their structures remain unclear and require future work to elucidate these analogues (See Supplementary, Figures S11–S13). Given that we are now expanding the structural diversity of the levesquamides, we have re-named levesquamide as levesquamide A (Figure 2).

## 2. Results & Discussion

### 2.1. Salt Screen for Levesquamide Production

**Figure 3:**
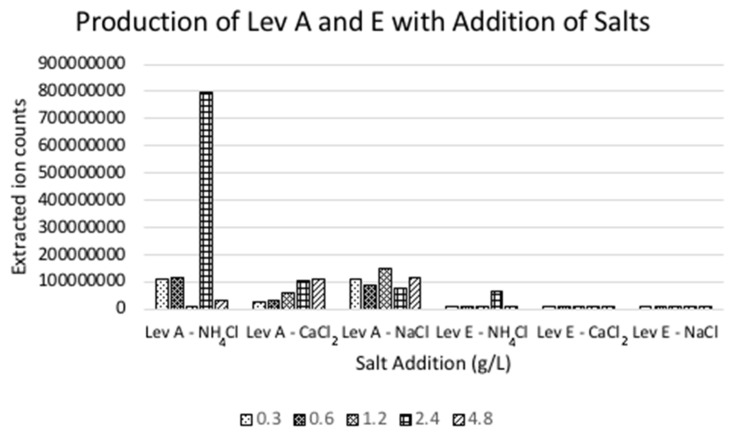
Integrated mass spectrometry peak areas of levesquamide A (Lev A) and levesquamide E (Lev E) in EtOAc extracts of fermentations performed in medium supplemented with NH_4_Cl, CaCl_2_ or NaCl at five concentrations (0.3, 0.6, 1.2, 2.4 and 4.8 g/L).

In order to elucidate the structure of the fused pyridine-isothioazolinone bicyclic ring system of levesquamide A, several stable isotope labeling studies were performed [10]. Re-analysis of MS^2^ data from the ^15^NH_4_Cl feeding study identified the generation of another compound which, through MS^2^ fragmentation was deemed to be a structural analogue of levesquamide (*m*/*z* 659.2151; levesquamide E). With this information at hand, a small salt screen was conducted to determine if other salts might upregulate production of levesquamide E and facilitate isolation of this new family member. Three salts (NH_4_Cl, CaCl_2_ and NaCl) were added individually to small-scale (10 mL) fermentations at five concentrations (0.3 g/L, 0.6 g/L, 1.2 g/L 2.4 g/L, and 4.8 g/L).Ethyl acetate (EtOAc) extracts of the fermentations were analyzed by UHPLC-HR-ESI-MS^+^ and the integrated extracted ion counts for levesquamide A ([M+H]^+^ *m*/*z* 631.2181) and E ([M+H]^+^ *m*/*z* 659.2151) were calculated to assess relative production levels in the different media (Figure 3).

Supplementation of media with NH_4_Cl at 2.4 g/L resulted in the strongest production of both levesquamide A and E. To generate sufficient extract to purify levesquamide E a scale-up fermentation was conducted (9 × 1 L) using the optimal NH_4_Cl concentration. Extraction of the scale-up fermentation with EtOAc yielded 1.6 g of crude extract.

### 2.2. Fractionation, Molecular Network Analysis and Isolation of Levesquamide B

**Figure 4:**
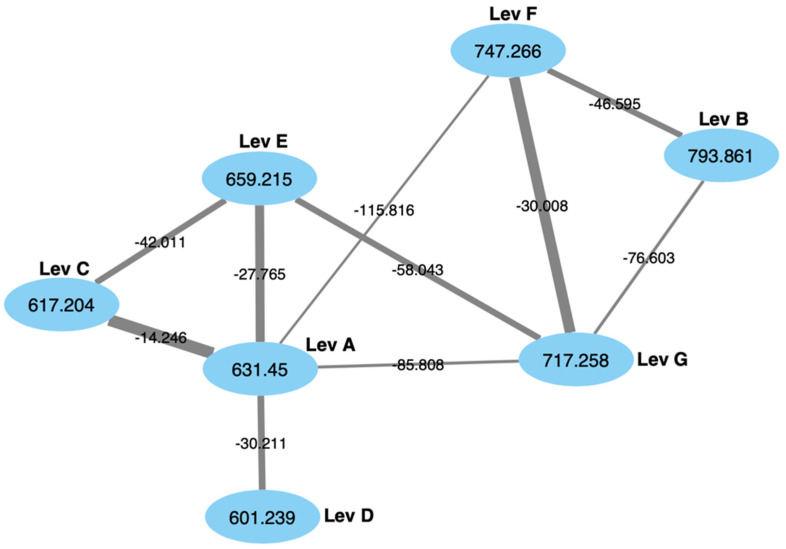
Global natural product social molecular networking cluster for the levesquamides, indicating a total of 7 family members, including levesquamide A (631), 6 analogues. Each node represents one ion, each is connected by an edge when the cosine score is ≥0.7, with the thickness of each edge representing the cosine score, and each edge is labelled with the *m*/*z* differences between the connecting nodes.

The crude extract was subjected to reverse-phase flash chromatography using a C_18_ stationary phase over a H_2_O:MeOH 95:5 to 2:98 gradient over 35 min. Levesquamide A (*m*/*z* 631.2181) eluted at 31 min and its analogue, levesquamide E (*m*/*z* 659.2151) eluted at 29 min. These fractions were subjected to analysis via UHPLC-HR-ESI-MS^+^. which led to the identification of another levesquamide analogue (*m*/*z* 793.2729), named levesquamide B. In order to determine how many levesquamides were present we conducted a molecular networking analysis using the Global Natural Product Social Molecular Networking (GNPS) server [15]. Within the molecular network, a 7-member cluster was identified as the levesquamide cluster (Figure 4). The cluster includes levesquamide A, and 6 additional levesquamides, B-G.

**Figure 5:**
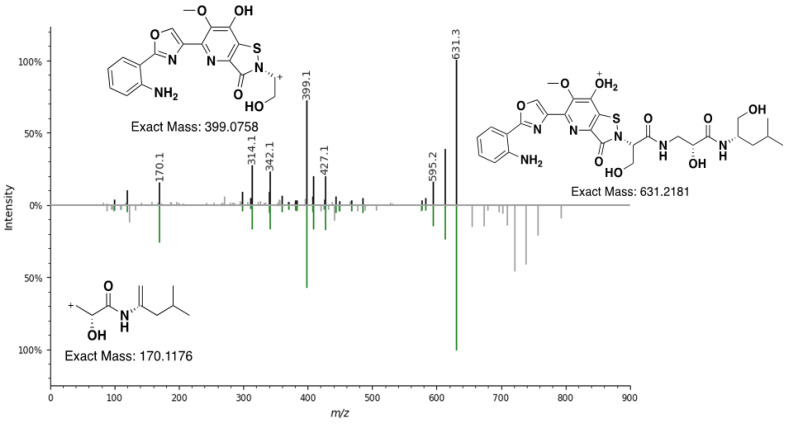
Mirror plot comparison of the MS^2^ fragmentation for Levesquamide A (**top**) and levesquamide B (**bottom**) including diagnostic fragments 399 and 170 *m*/*z* fragments.

To confirm the correct annotation of each ion, the MS^2^ fragmentation patterns were analyzed manually for similarities to levesquamide A fragments (see Figure 5 and Figures S7–S12). Extracted ion counts for the analogues were integrated to identify the relative abundance in order to prioritize analogs for purification. Levesquamide B had the next highest production level to levesquamide A and as such, we set out to purify this analog instead of the initially identified levesquamide E. Mass-guided RP-HPLC was performed with levesquamide A and levesquamide B eluting at 25 min and 19 min, respectively.

### 2.3. Structural Elucidation of Levesquamide B

Previous work in our group identified the BGC for the levesquamides. Genes encoding for the incorporation of L-serine (module 5) and L-leucine (module 7, reduced to leucinol by the terminal reductase domain) were readily identified, while D-isoserine was predicted to arise from hydroxylation of the achiral β-alanine residue incorporated into levesquamide by module 6. The stereochemistry of these residues was determined through the elucidation of the levesquamide A structure, supported by analysis of the BGC which did not reveal the presence of epimerization domains responsible for catalysing epimerization of amino acids in NRP biosynthesis [11].

MS^2^ analysis of levesquamide B displayed initial fragmentation down to the mass of levesquamide A (*m*/*z* 631.2181) indicating the addition of a moiety at a primary alcohol, susceptible to fragmentation. Comparison of chemical shifts between levesquamide B and levesquamide A were in good agreement for the anthranilate, oxazole, serine, isoserine and leucinol residues. 2D-NMR experiments presented almost identical coupling patterns across COSY, HSQC and HMBC spectra to previously reported data [10]. Minor chemical shift changes of the MeO-Ac2 (3.97 LevA to 3.90 LevB) provided insight to the modification being located within the pyrimidine ring. The levesquamides possess few protons across the fused pyridine−isothiazolinone moiety making NMR analysis problematic and as such we decided to carefully examine the MS^2^ fragmentation. The loss of 162 *m*/*z* was observed, and in combination with increasing compound polarity of levesquamide B compared to levesquamide A, led us to consider the possibility of the addition of glucose or galactose on the pyrimidine ring. This hypothesis was further supported through the addition of ^1^H-NMR signals between 3–4.5 ppm. At 4.25 ppm a doublet could be observed integrating to a single proton whilst HSQC correlations were observed at 98.1 ppm, indicative of an anomeric carbon (Table 1).

**Figure 6:**
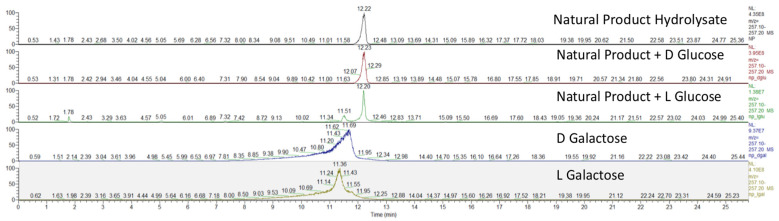
Extracted ion count for tanakas sugar stereochemical conformation displaying the natural product hydrolysate to have identical retention time to D-glucose.

To test our hypothesis that the glycosyl group either glucose or galactose, levesquamide B was subjected to hydrolysis and derivatization using Tanaka’s method [16]. Standards of both L and D—glucose and galactose were derivatized under identical conditions. Due to similar retention times, the natural product hydrolysate was analyzed alone before a co-injection with D and L glucose, respectively. The co-injection of the natural product hydrolysate and D-glucose derivative presented a single peak. Upon co-injection with the L-glucose derivative an earlier peak at a retention time of 11.51 could be observed within the extracted ion count. Both D and L galactose derivatives eluted earlier than the natural product with retention times of 11.69 and 11.36, respectively. From this we conclude that the glycoside present within levesquamide B to be D-glucose (Figure 6).

In order to find optimal production of levesquamide B a a comprehensive media screen of 30 media was performed.Each media was extracted with EtOAc and extracted ion counts analysed to compare production levels. Production of levesquamide B was only observed in BFM23m supplemented with NH_4_Cl. Interestingly it’s producion was dependent on the presence of NH_4_Cl as the compound was not detected in BFM23m lacking this ingredient.

### 2.4. MS^2^ Analysis of Other Levesquamide Analogues

Through the use of MS^2^ fragmentation we also determined the putative structures of levesquamides C and D. Levesquamide C differed by a *m*/*z* of 14 with the diagnostic fragment of 399 *m*/*z*, indicative of the preservation of the polycyclic portion of the molecule. With the 399 *m*/*z* fragment retained it was concluded that the alteration must be localized to the amino acid terminus. With a loss of 14 *m*/*z* and a new lower mass fragment of 156 *m*/*z* now present within the MS^2^ fragmentation data, the loss of CH_2_ in the terminal amino acid was deemed likely. This difference is consistent with the incorporation of a valine instead of leucine by the NRPS module 7 of the levesquamide BGC. Substrate flexibility of NRPS domain incorporating hydrophobic amino acids (Leu, Ile, Val) is a common feature in peptide biosynthesis, often resulting in the production of multiple peptide analogs. Following incorporation of a residue instead of a leucine the terminal reductase domain would release the peptide as an alcohol with a terminal valinol residue giving rise to the final structure of levesquamide C. Levesquamide D was found to have an M+H^+^ of 601.2641. With smaller mass fragments of 170 *m*/*z* preserved, as well as the fragments of the amino acid tail of the molecule identical to that of levesquamide A, we believe the alteration to be a loss of the methoxy located on the hydroxypyridine ring. Interestingly, within our previous publication we found LvqF to be responsible for methylation and disruption led to abolishment of levesquamide production [10]. As such this suggests that either methylation occurs early in the biosynthesis or there was a polar effect on the down stream genes. As both levesquamide C, and levesquamide D (loss of OCH_3_) are seen within the molecular network it is likely polar effects lead to inactiveation of the levesquamide cluster

## 3. Experimental

### 3.1. General Experimental Procedures

A Thermo Scientific Vanquish UHPLC chromatograph equipped with HRMS-CAD-UV detection, which included a Thermo Scientific ID-X Tribrid mass spectrometer fitted with a heated electrospray ionization (H-ESI) source, a Thermo Scientific charged aerosol detector VF-D20-A, and a Thermo Scientific diode array detector (DAD) VF-D11-A-01 scanning 200 nm–600 nm was used. The solvents A = 0.1% formic acid (FA) in H_2_O and B = 0.1% FA in acetonitrile were used at a 0.5 mL/min flow rate with a Kinetex 1.7 μm C18 100 Å (50 × 2.1 mm) with the following gradient: 0 min = 5% B, 0.2 min = 5% B (isocratic), 4.8 min = 98% B, 8 min = 98% B (isocratic), 8.5 min = 5% B, 9.8 min = 5% B (isocratic). The MS parameters include positive ion scans performed from 150–2000 amu at an ion transfer tube temperature of 300 °C and a vaporizer temperature of 275 °C.

NMR spectra were obtained on a Bruker Avance III NMR spectrometer (1H: 600 MHz, 13C: 151 MHz) equipped with a 5 mm cryoprobe. All chemical shifts (δ) are referenced to the DMSO-d6 residual solvent peaks [1H (DMSO-d6): 2.50 ppm; 13C (DMSO-d6): 39.51 ppm].

Automated flash chromatography was performed on a Teledyne Combiflash Rf200 using C18 RediSep columns (24 g). HPLC purifications were carried out on a Waters auto purification system coupled with an evaporative light-scattering detector and UV detector. All amino acid standards were purchased from Sigma-Aldrich. All reagents were purchased from commercial sources and used without further purification unless otherwise stated.

### 3.2. Salt Screen

A two-stage seed culture process was used to generate inoculum. The strain wasmaintained on marine ISP3m (ISP3m; ISP3 supplemented with 18 g/L Instant Ocean^®^, Spectrum Brands) agar plates incubated at 30 °C. Approximately 1 cm^2^ of the agar culture was used to inoculate 7 mL of ISP2m broth in a 25 × 150 mm culture tube containing five 4 mm glass beads and incubated at 30 °C and 200 RPM. After 72 h, 1 mL of the first-stage seed was transferred to 7 mL of fresh ISP2m broth and incubated under the same conditions for 24 h. After 24 h 0.5 mL of the second stage seed as transferred into triplicate 10 mL fermentations of RKND-216 performed in BFM23m base medium and variations containing different salts (NaCl, CaCl_2_ or NH_4_Cl) added at five different concentrations (0.3, 0.6, 1.2, 2.4 and 4.8 g/L). After incubating for 10 days at 30 °C with shaking at 200 RPM, fermentations were extracted with an equal volume of EtOAc. The extracts were dried *in vacuo* and dissolved in 1 mL of MeOH and subjected to UHPLC-HR-ESI-MS analysis.

### 3.3. Large Scale Fermentation and Extraction

A two-stage seed culture process was performed as previously described above. The second-stage seeds from multiple tubes were combined, and 30 mL of seed culture was used to inoculate each of 9 Fernbach flasks containing 1 L of fresh BFM23m supplemented with 2.4 g/L NH_4_Cl (BFM23m; BFM23 supplemented with 18 g/L Instant Ocean^®^) broth each. After incubating for 10 days at 30 °C with shaking at 200 RPM, the culture was extracted three times with equal volumes of EtOAc. The organic layers were combined and dried *in vacuo,* providing 1.6 g of crude extract

### 3.4. Global Natural Product Social Networking (GNPS) Analysis of Family Members

The acquired UHPLC-MS/MS chromatograms were converted from a .RAW file to an open source MS file type .mzML using the open source GUI msConvert (ver. 3.0.18232), part of the ProteoWizard tool kit [17]. The .mzML file was uploaded to the GNPS server via using WinSCP (https://winscp.net/eng/download.php (accessed on 14 March 2022)). The molecular network was generated using Global Natural Products Social Molecular Networking [15]. The spectral analysis and specific network generation parameters can be found following the job link (https://gnps.ucsd.edu/ProteoSAFe/status.jsp?task=c18a7718fb9c40b0b56d95db62eef1c9 (accessed on 12 September 2022)). The MS/MS data were deposited in the MassIVE Public GNPS data sets (https://massive.ucsd.edu (accessed on 12 September 2022)) with the accession no. MSV00009031. Notable molecular network setting parameters were as follows: precursor ion mass tolerance 2.0; fragment ion mass tolerance, 0.5; minimum pairs cos, 0.7; network TopK, 10; minimum matched peaks, 6; minimum cluster size 2. The molecular network was analyzed and visualized using Cytoscape (ver. 3.8.1) [18]. The molecular network cluster contained 6 unknowns all clusters containing the previously isolated levesquamide A [10]. The analogues were then reanalysed through the use of Xcalibur (Thermo Scientific, Waltham, MA, USA; version 4.2) and their MS^2^ fragmentation patterns compared to that of levesquamide A giving arise to a total of 7 compounds within the levesquamide family.

### 3.5. Chromatographic Purification

The entire *Streptomyces* sp. RKND-216 crude extract was prepared for solid load injection by adsorbing on C18 and the separation was performed with a 130 g C18 column (RediSep Rf) using a mobile phase flow rate of 32 mL/min. The mobile phase consisted of a linear gradient from H2O:MeOH (95%:5%) to 100% MeOH over 30 min followed by 100% MeOH for 5 min. Levesquamide B was further purified RP-HPLC using a semi-preparative Luna C18 100 Å column (250 × 10 mm, 5 μm; Phenomenex, Torrance, CA, USA, 213436-1). An isocratic elution with 35% H2O containing 0.1% formic acid and 65% MeOH containing 0.1% FA was used over 30 min. The eluent was monitored by evaporative light scattering detection, and UV at 277 and 348 nm while levesquamide B was collected at 19 min. Subsequent evaporation *in vacuo* resulted in 0.75 mg of pure levesquamide B.

### 3.6. Glycoside Stereochemical Analysis by Tanaka’s Method [16]

Levesquamide B (0.25 mg) was dissolved in 200 µL 2 M HCl and heated to 110 °C for 16 h before allowing to return to ambient temperature and evaporated to dryness *in vacuo*. The sample was re-suspended in 0.5 mL of pyridine followed by 0.5 mL of L-cysteine methyl ester stock (5 mg/mL) before heating for 1 h at 60 °C. After 1 h, 0.5 mL of *o*-tolyl isothiocyanate in pyridine (5 mg/mL) was added and the reaction was again heated to 60 °C for 1 h. The reaction was concentrated under reduced pressure and resuspended in MeOH for analysis via UHPLC-HR-ESI-MS. This process was repeated for all glycoside standards.

Tanakas method was conducted using a Phenomenex Kinetex PFP 100 Å C_18_ (4.6 × 150 mm, 2.6 μm); mobile phase flow rate, 0.8 mL·min^−1^; injection volume, 10 μL; linear gradient, H_2_O:MeOH (95:5, 0.1% FA) at 0.5 min to H_2_O:MeOH (40:60, 0.1% FA) at 13.0 min, which was held until 15.0 min before another linear gradient to 100% MeOH (0.1% FA) at 17.0 min, which was held until 20.0 min before returning to H_2_O:MeOH (87:13, 0.1% FA) at 20.1 min and equilibrating for 5.0 min. The following HRMS parameters were used: positive ionization mode; mass resolution, 30,000; mass range, *m*/*z* 190 to 2000; spray voltage, 2.0 kV; capillary temperature, 300 °C; S-lens RF voltage, 60.0%, maximum injection time, 10 ms; 1 microscan.

## 4. Conclusions

Reinvestigation of RKND-216 has demonstrated new natural products can still be attainted through currently known microbes. The levesquamides have the potential to be an important group of compounds with levesquamide A presenting promising antitubercular properties. With technological tools continuing to advance so does our biodiscovery capabilities. Using GNPS we were able to identify six new members the levesquamide family and through the use of spectroscopical methods, we have elucidated the structure of levesquamide B. We have also presented the putative structures of levesquamide C and D, along with the MS^2^ fragmentation data for levesquamides E, F and G. Further work must be conducted in order to determine their structures whilst exploring the antimicrobial activity of the newly described analogues.

## Figures and Tables

**Table 1:** NMR spectroscopic data (600 MHz dimethyl sulfoxide (DMSO)- d6) for levesquamide B.

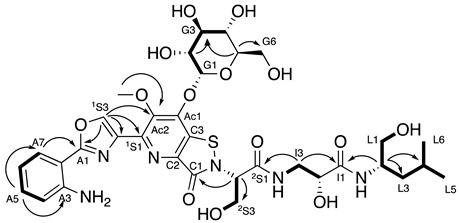					
	Position	*δ*_C/N_, Type	*δ*_H_, Mult. (*J* in Hz)	COSY	HMBC
Anthranilate	A1	160.6, C	-	-	-
	A2	106.6, C	-		-
	A3	148.2, C	-	-	-
	A4	115.8, CH	6.87, dd (1.1, 8.3)	A5	A6, A2, A1, A3-NH_2_
	A5	130.8, CH	7.20, ddd (7.1, 8.5, 1.6)	A4, A6	A3, A7
	A6	115.0, CH	6.65, dd (8.1, 7.0)	A5, A7	A4, A5, A2, A7
	A7	126.9, CH	7.75, dd (1.0, 7.6)	A6	A3, A5, A1
	NH_2_-A3	64.5, NH_2_	7.00, s	-	A4
^1^Serine	^1^S1	158.4, C	-	-	-
	^1^S2	131.2, C	-		-
	^1^S3	137.2, CH	8.40, s	-	A1, ^1^S2, ^1^S1, ^1^S2-N
	N-^1^S2	236.4, N	-	-	-
Acetate	Ac1	162.4, C	-	-	-
	Ac2	146.0, C	-	-	-
	MeO-Ac2	57.5, CH_3_	3.90, s	-	Ac2
Glucose	G1	92.6, CH	4.21, d (3.6)	G2	
	G2	73.5, CH	3.22, m	G1,G2	G3
	G3	72.1,	3.31, m	G2,G4	
	G4	73.5,	3.34, m	G3	
	G5	70.6	3.45, m		G3,G6
	G6	61.1 CH_2_	3.81, m		G5
Cysteine	C1	166.1, C	-	-	-
	C2	134.9, C	-	-	-
	C3	132.0, C	-	-	-
	N-C2	nd	-	-	-
^2^Serine	^2^S1	167.6, C	-	-	-
	^2^S2	58.1, CH	5.19, t (5.7)	^2^S3	C2^c^, C1, ^2^S3
	^2^S3	61.1, CH_2_	3.82, m	^2^S2, ^2^S3-OH	^2^S2, ^2^S1
	OH-^2^S3	-	5.32, broad	^2^S3	-
	N-^2^S2	125.6, N	-	-	-
Isoserine	I1	171.4, C	-	-	-
	I2	70.1, CH	3.96, m	I3, I2-OH	I3, I1
	I3a	43.4, CH_2_	3.46, m	I2, I3b, I3-NH	^2^S1, I2, I1
	I3b		3.12, m	I2, I3a, I3-NH	
	OH-I2	-	5.74, d (5.1)	I2	I3, I2, I1
	NH-I3	111.6, NH	8.43, t (5.7)	I3	I3, ^2^S2, I2, ^2^S1
Leucinol	L1a	63.7, CH_2_	3.36, m	L1b, L2, L1-OH	L3, L2
	L1b		3.28, m	L1a, L2, L1-OH	
	L2	48.4, CH	3.81, m	L1, L3, L2-NH	L4, L1, L3, I1
	L3	40.0, CH_2_	1.31, m	L2, L4	L2, L4, L5, L6, L1
	L4	24.2, CH	1.55, m	L3, L5, L6	L2, L3, L5, L6
	L5	23.4, CH_3_	0.86, d (6.7)	L4	L6, L3
	L6	21.2, CH_3_	0.83, d (6.5)	L4	L5, L3
	OH-L1	-	4.67, s	L1	-
	NH-L2	121.5, NH	7.38, d (8.8)	L2	L3, L2, L1, I2, I1

## Data Availability

Not Applicable.

## Supplementary Materials

The following supporting information can be downloaded at: https://www.mdpi.com/article/10.3390/molecules27227794/s1. For levesquamide B be spectroscopic data includes 1H NMR, COSY, HSQC, HMBC, 13C NMR as well as HRMS and MS2 fragmentation patterns. For other levesquamide analogues HMRS and MS2 fragmentation patterns are available.
