# Supplementary material for: Discovery of Levesquamide B through Global Natural Product Social Molecular Networking
Source: Molecules. 2022 Nov 12;27(22):7794. doi: 10.3390/molecules27227794 (PMC9695545; doi:10.3390/molecules27227794)
Supplement: Supplementary file 1 [file molecules-27-07794-s001.zip › molecules-1994186-supplementary.pdf]

Supplementary information

Discovery of Levesquamide B through Global Natural Product Social Molecular Networking

Mary M. LeClair [1,†], Zacharie A. Maw [2], Alyssa L. Grunwald [3], Joshua R. Kelly [3], Bradley A. Haltli [2,3], Russell G. Kerr [1,2,3] and Christopher Cartmell [1,4,†,*]

1 Department of Chemistry, University of Prince Edward Island, 550 University Avenue, Charlottetown, PE C1A 4P3, Canada
2 Department of Biomedical Sciences, Atlantic Veterinary College, 550 University Avenue, Charlottetown, PE C1A 4P3, Canada
3 Nautilus Biosciences Croda, 550 University Avenue, Charlottetown, PE C1A 4P3, Canada
4 Antimicrobial Discovery Center, Department of Biology, Northeastern University, Boston, MA 02115, USA
† These authors contributed equally to this work.
* Correspondence: ccartmell@upei.ca or c.cartmell@northeastern.edu

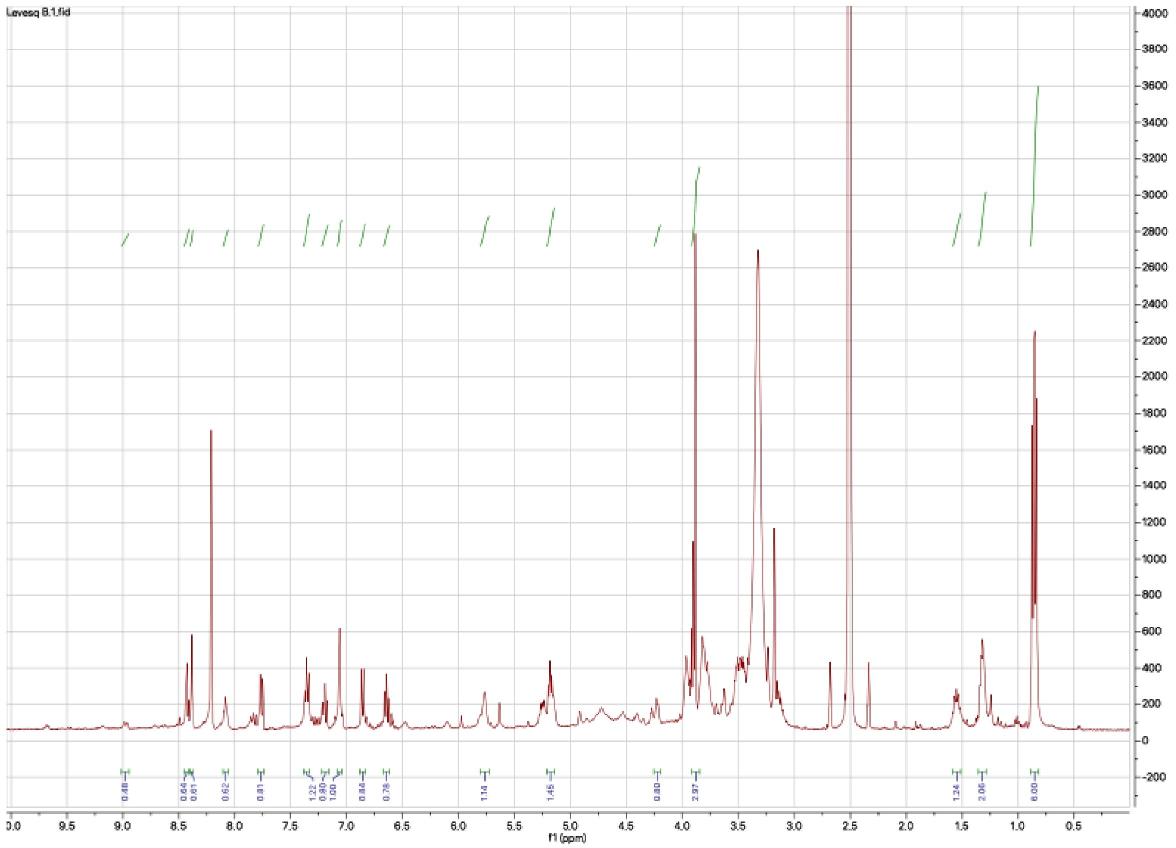

Figure S1: 1H NMR of Levesquamide B

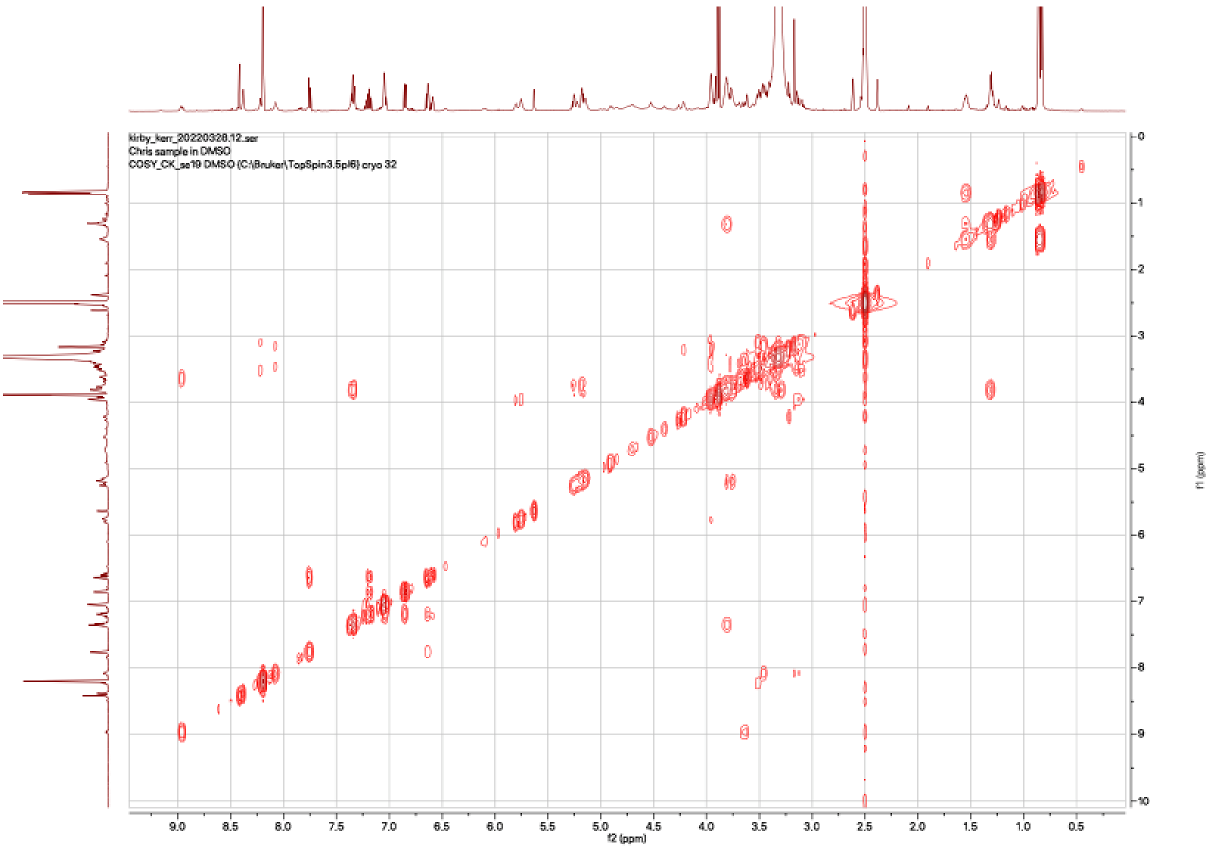

Figure S2: COSY NMR of Levesquamide B

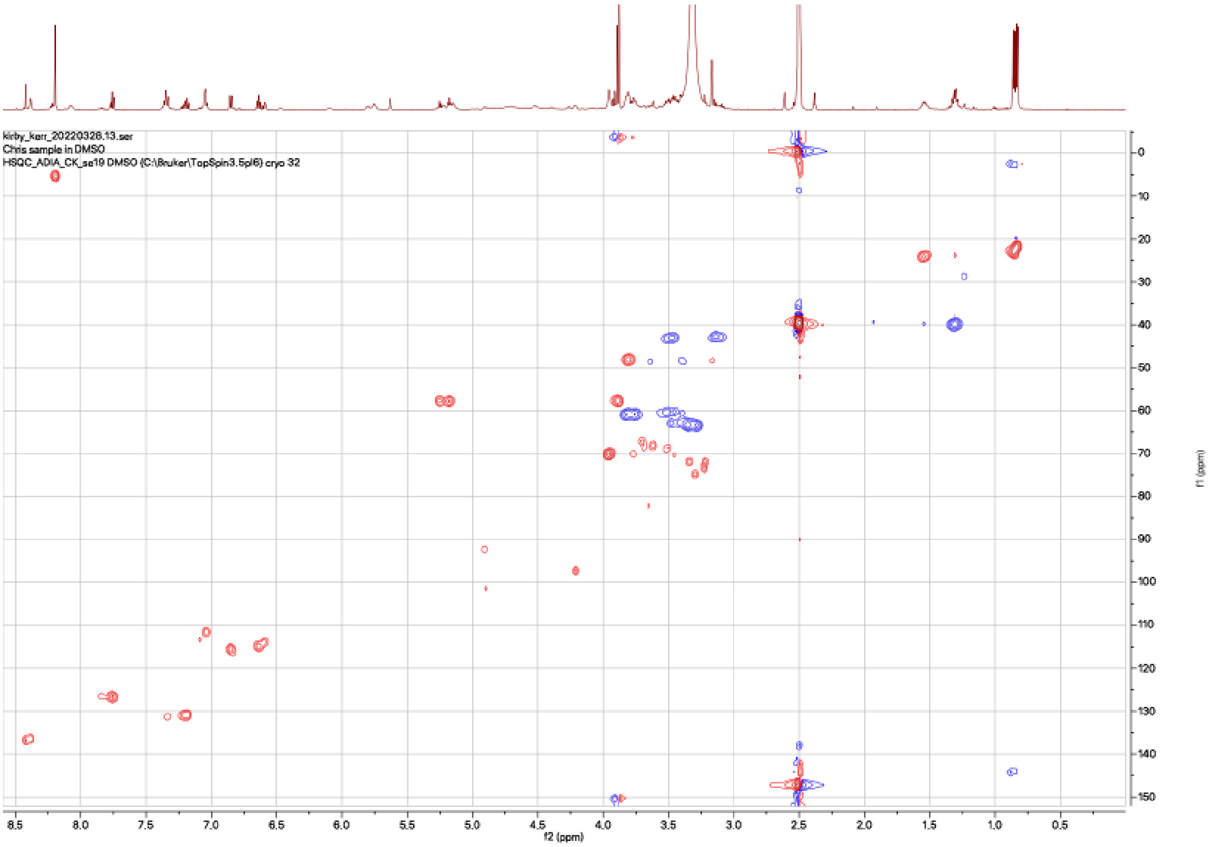

Figure S3: HSQC NMR of Levesquamide B

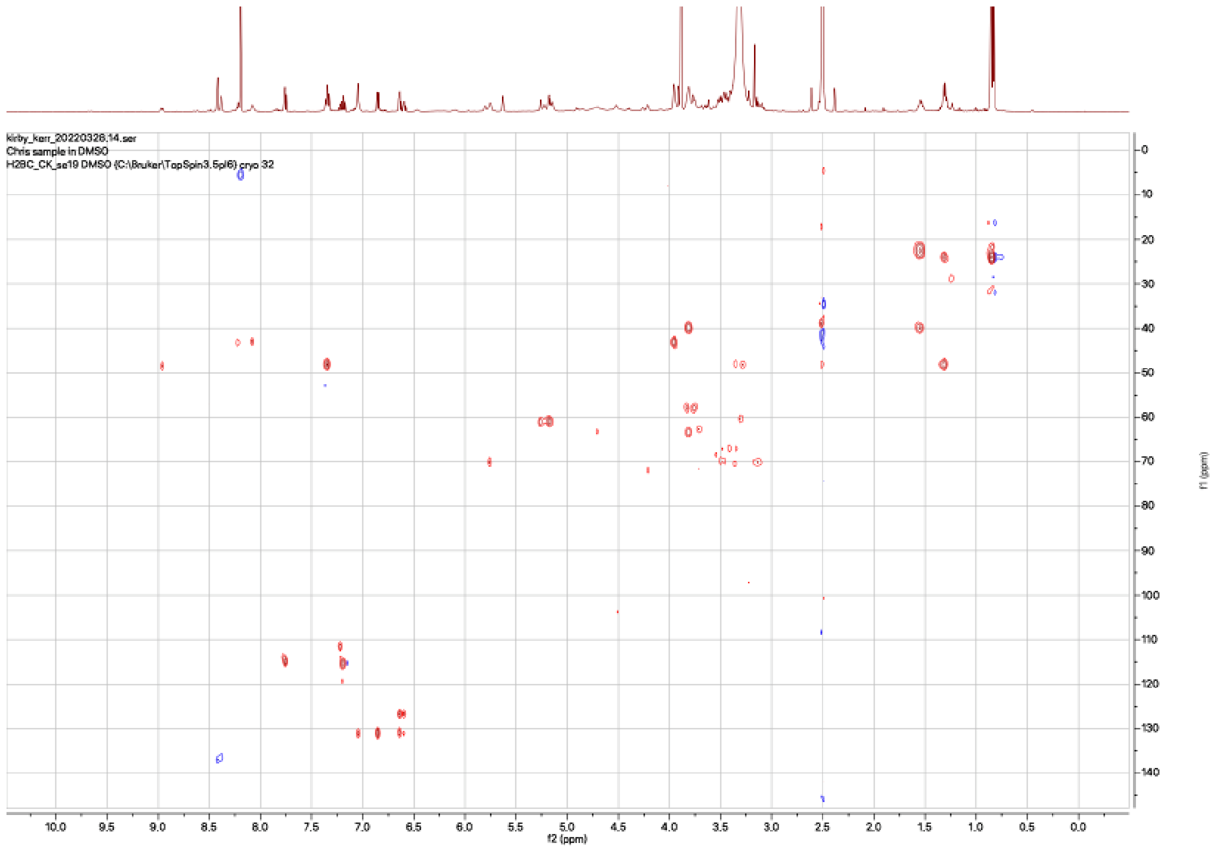

Figure S4: H2BC NMR of Levesquamide B

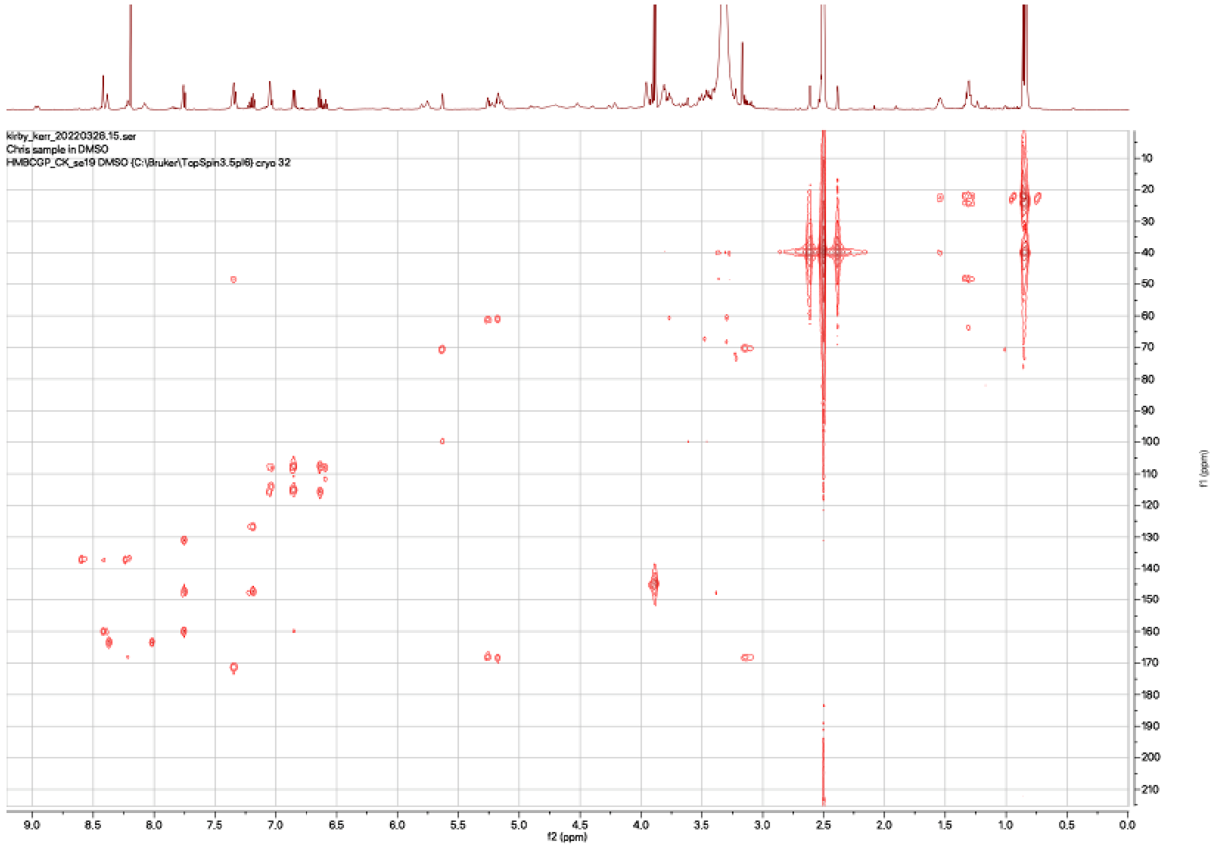

Figure S5: HMBC NMR of Levesquamide B

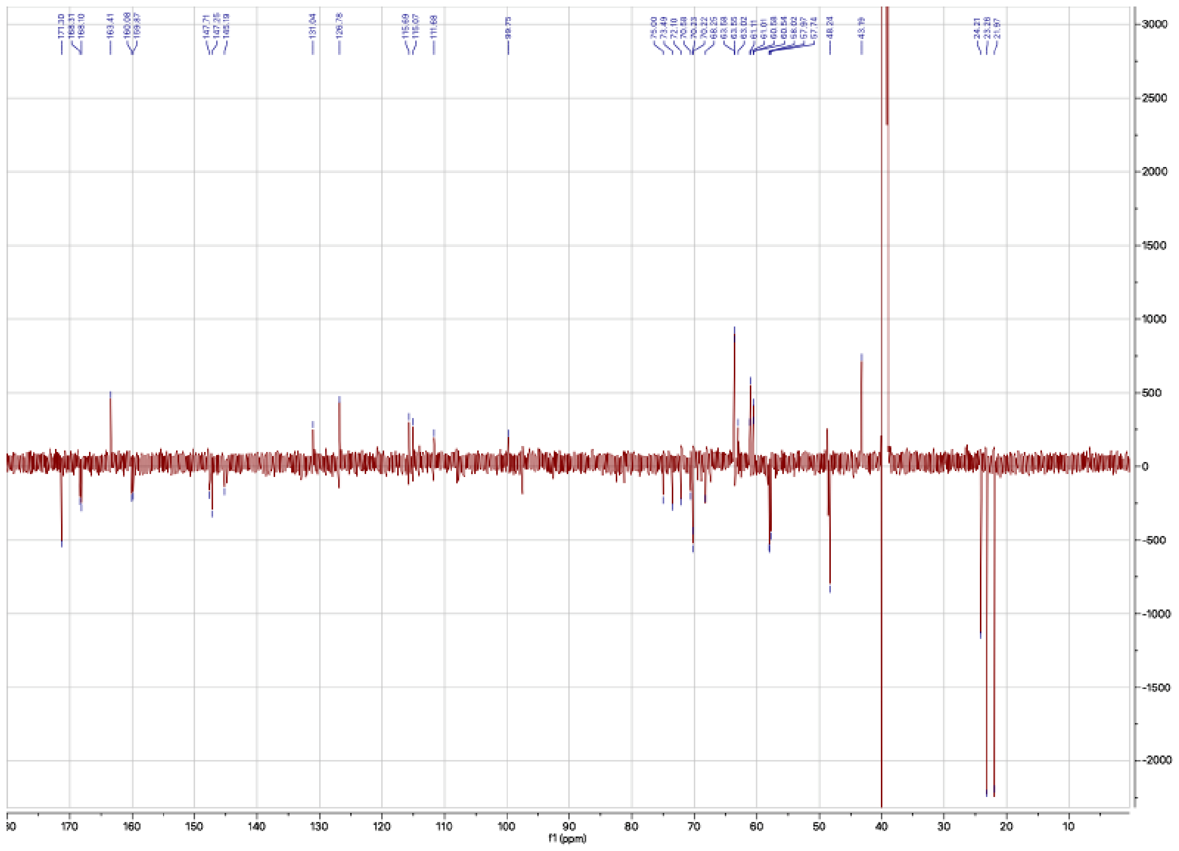

Figure S6: DEPTq NMR of Levesquamide B

Levesquamide A

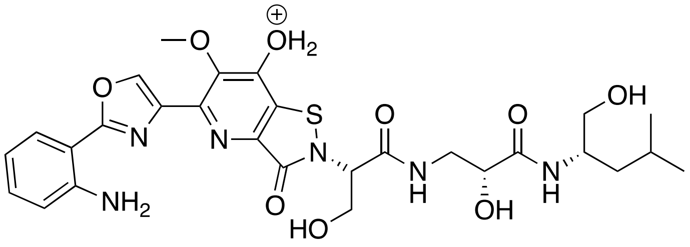

Chemical Formula: $C_{28}H_{35}N_6O_9S^+$
Exact Mass: 631.2181

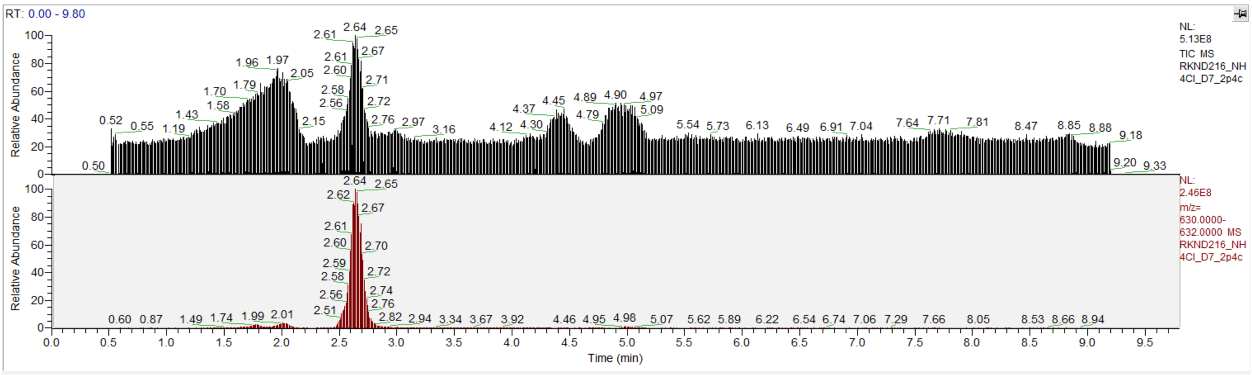

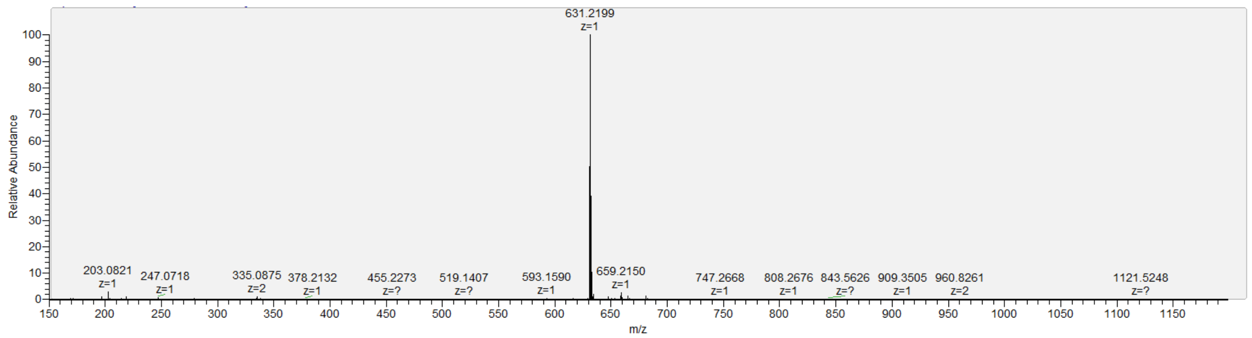
631.2199
z=1
203.0821
z=1
247.0718
z=1
335.0875
z=2
378.2132
z=1
455.2273
z=?
519.1407
z=?
593.1590
z=1
659.2150
z=1
747.2668
z=1
808.2676
z=1
843.5626
z=?
909.3505
z=1
960.8261
z=2
1121.5248
z=?
Relative Abundance
m/z

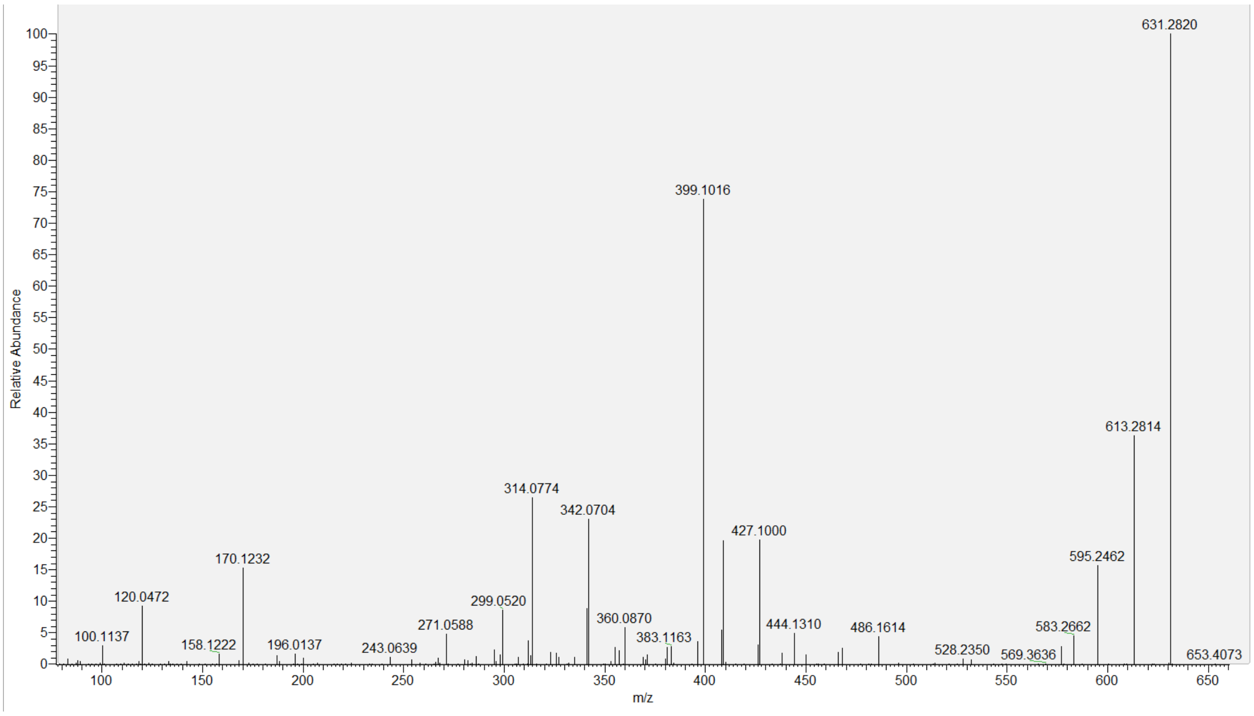
631.2820
399.1016
613.2814
314.0774
342.0704
427.1000
170.1232
595.2462
120.0472
299.0520
271.0588
360.0870
444.1310
486.1614
583.2662
100.1137
383.1163
158.1222
196.0137
243.0639
528.2350
569.3636
653.4073
Relative Abundance
m/z

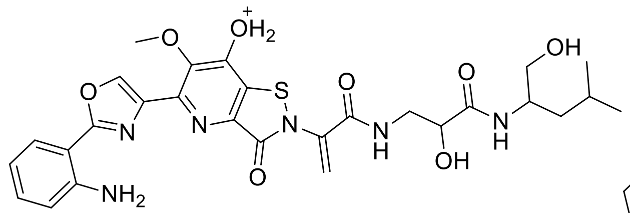

Chemical Formula: $C_{28}H_{33}N_6O_8S^+$
Exact Mass: 613.20751

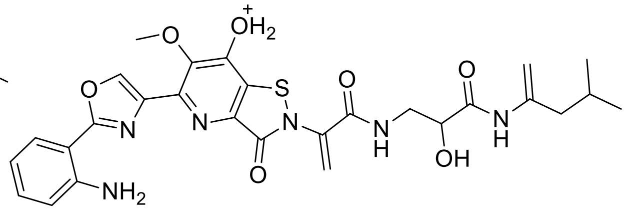

Chemical Formula: $C_{28}H_{31}N_6O_7S^+$
Exact Mass: 595.19694

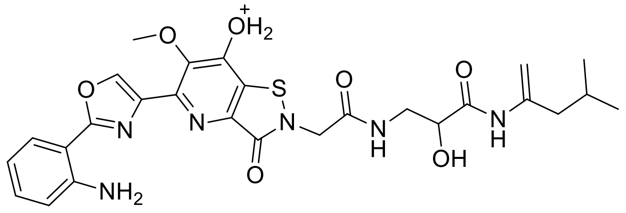

Chemical Formula: $C_{27}H_{31}N_6O_7S^+$
Exact Mass: 583.19694

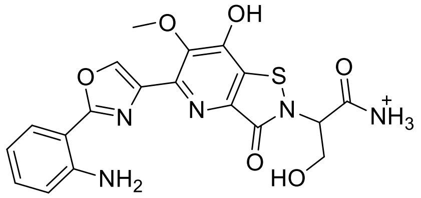

Chemical Formula: $C_{19}H_{18}N_5O_6S^+$
Exact Mass: 444.09723

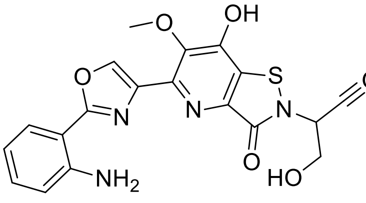

Chemical Formula: $C_{19}H_{15}N_4O_6S^+$
Exact Mass: 427.07068

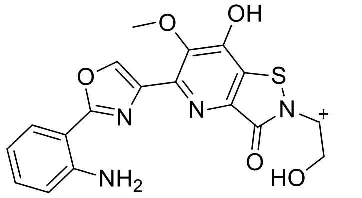

Chemical Formula: $C_{18}H_{15}N_4O_5S^+$
Exact Mass: 399.07577

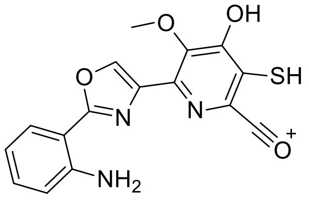

Chemical Formula: $C_{16}H_{12}N_3O_4S^+$
Exact Mass: 342.05430

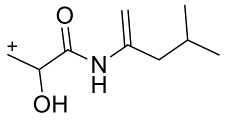

Chemical Formula: $C_9H_{16}NO_2^+$
Exact Mass: 170.11756

Figure S7: Structure of levesquamide A (A). TIC and EIC of levesquamide A (B). MS^1 spectra for levesquamide A (C). MS^2 fragmentation spectra for levesquamide A (D). Predicted observed fragments for levesquamide A (E).

Levesquamide B

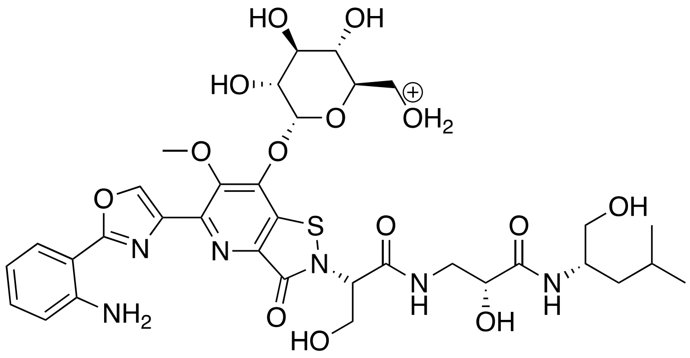

Chemical Formula: $C_{34}H_{45}N_6O_{14}S^+$
Exact Mass: 793.2709

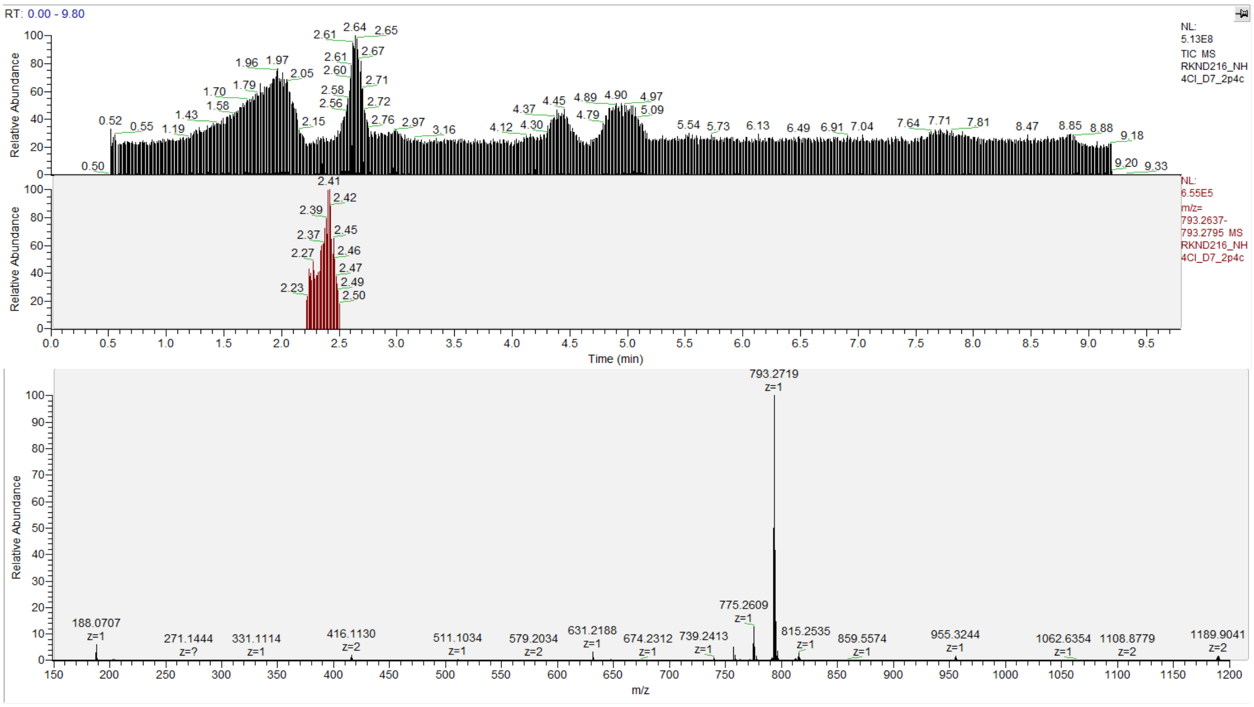

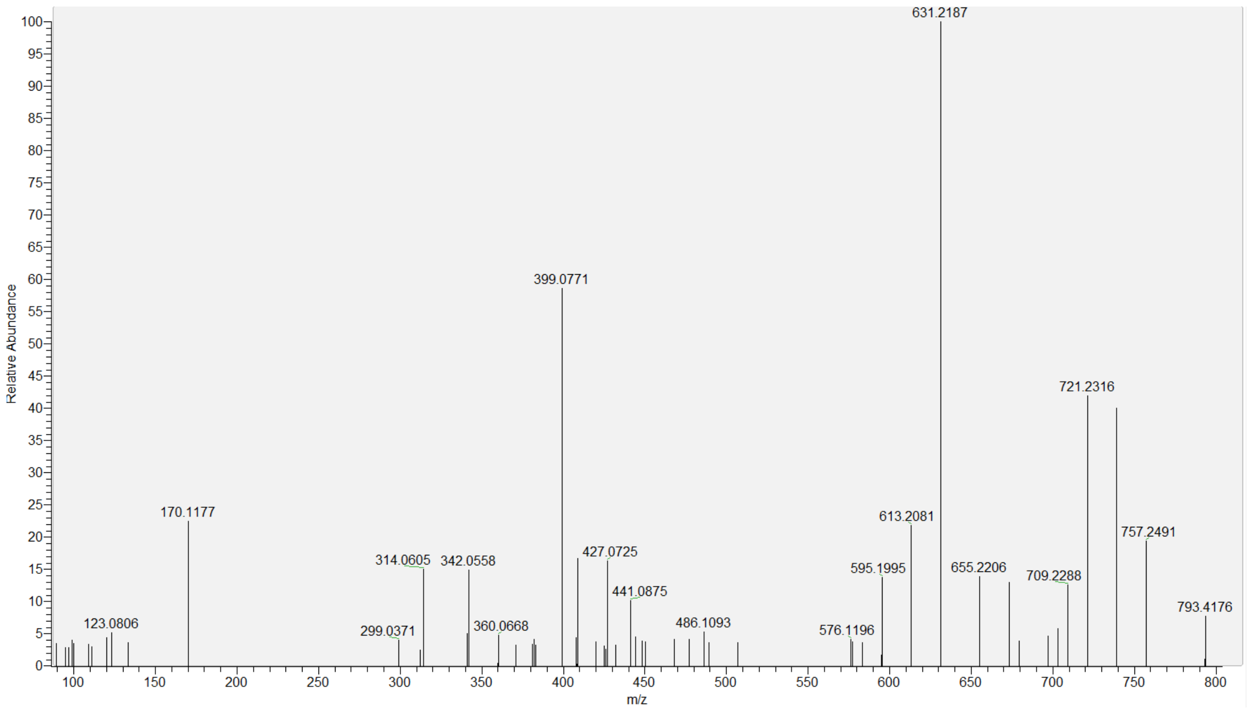

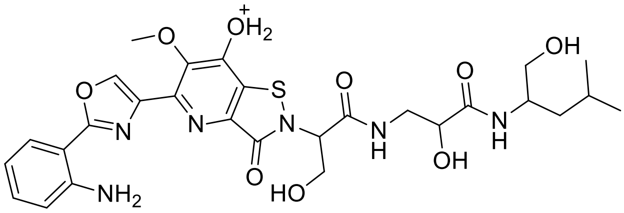

Chemical Formula: $C_{28}H_{35}N_6O_9S^+$
Exact Mass: 631.21807

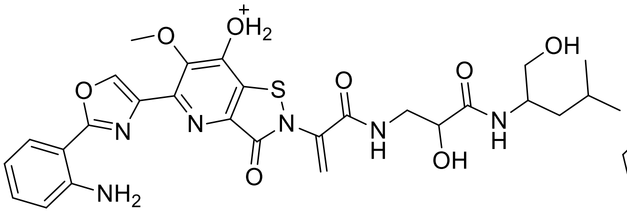

Chemical Formula: $C_{28}H_{33}N_6O_8S^+$
Exact Mass: 613.20751

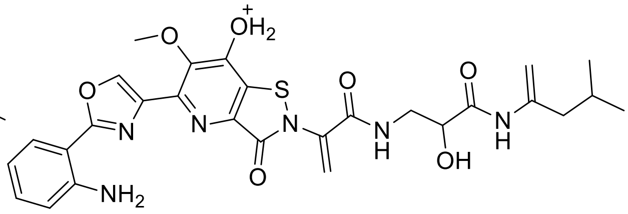

Chemical Formula: $C_{28}H_{31}N_6O_7S^+$
Exact Mass: 595.19694

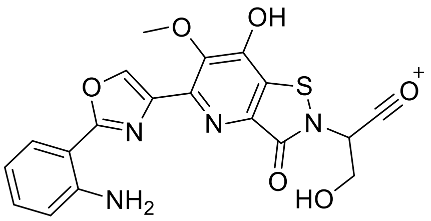

Chemical Formula: $C_{19}H_{15}N_4O_6S^+$
Exact Mass: 427.07068

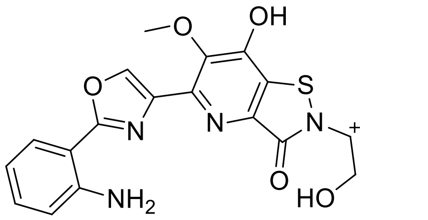

Chemical Formula: $C_{18}H_{15}N_4O_5S^+$
Exact Mass: 399.07577

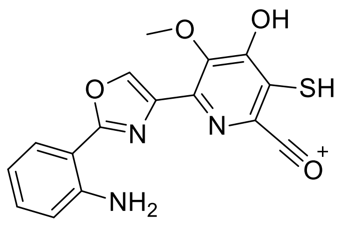

Chemical Formula: $C_{16}H_{12}N_3O_4S^+$
Exact Mass: 342.05430

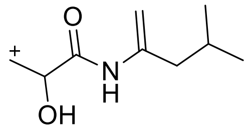

Chemical Formula: $C_9H_{16}NO_2^+$
Exact Mass: 170.11756

Figure S8: Structure of levesquamide B (A). TIC and EIC of levesquamide B (B). MS^1 spectra for levesquamide B (C). MS^2 fragmentation spectra for levesquamide B (D). Predicted observed fragments for levesquamide B (E).

Levesquamide C – putative structure [M+H] 617.2041 *m/z*, (Leucinol→Valinol).

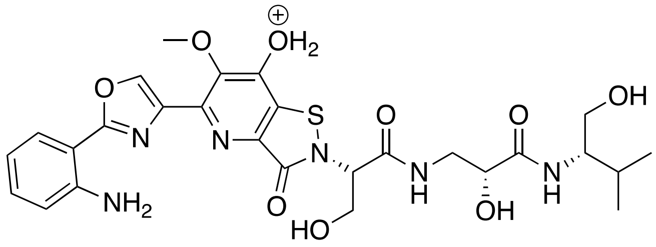

Chemical Formula: $C_{27}H_{33}N_6O_9S^+$
Exact Mass: 617.2024

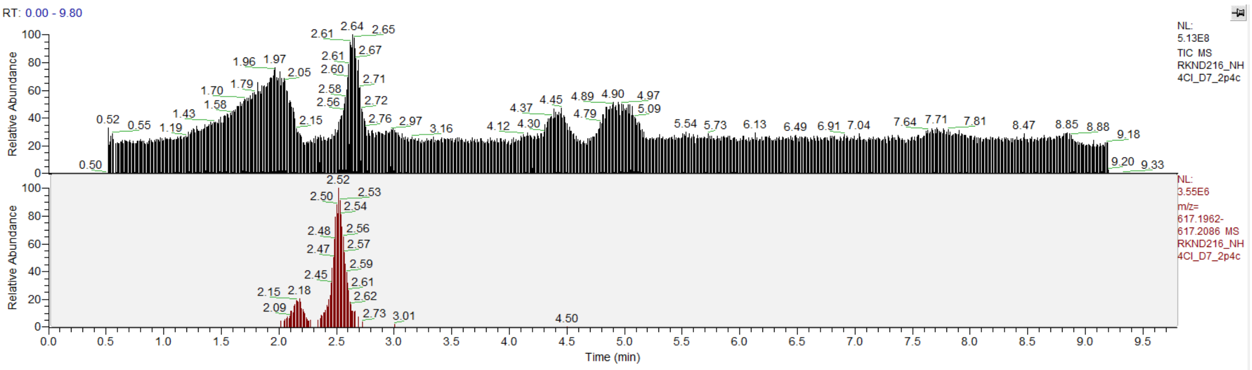

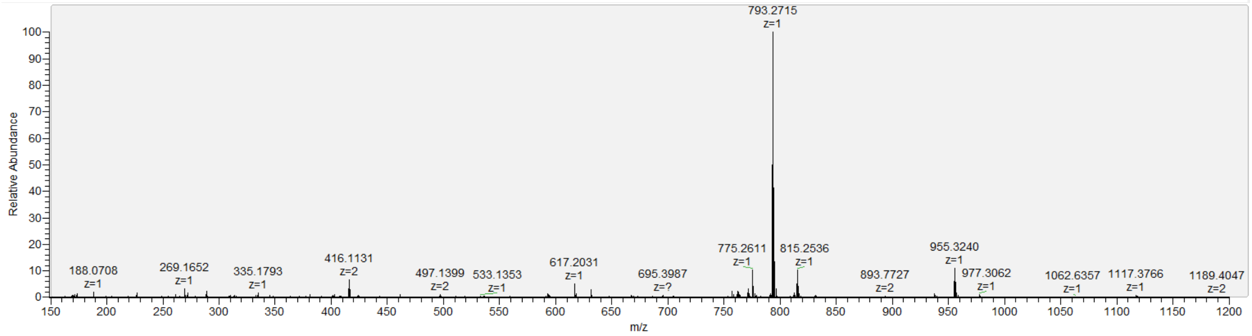

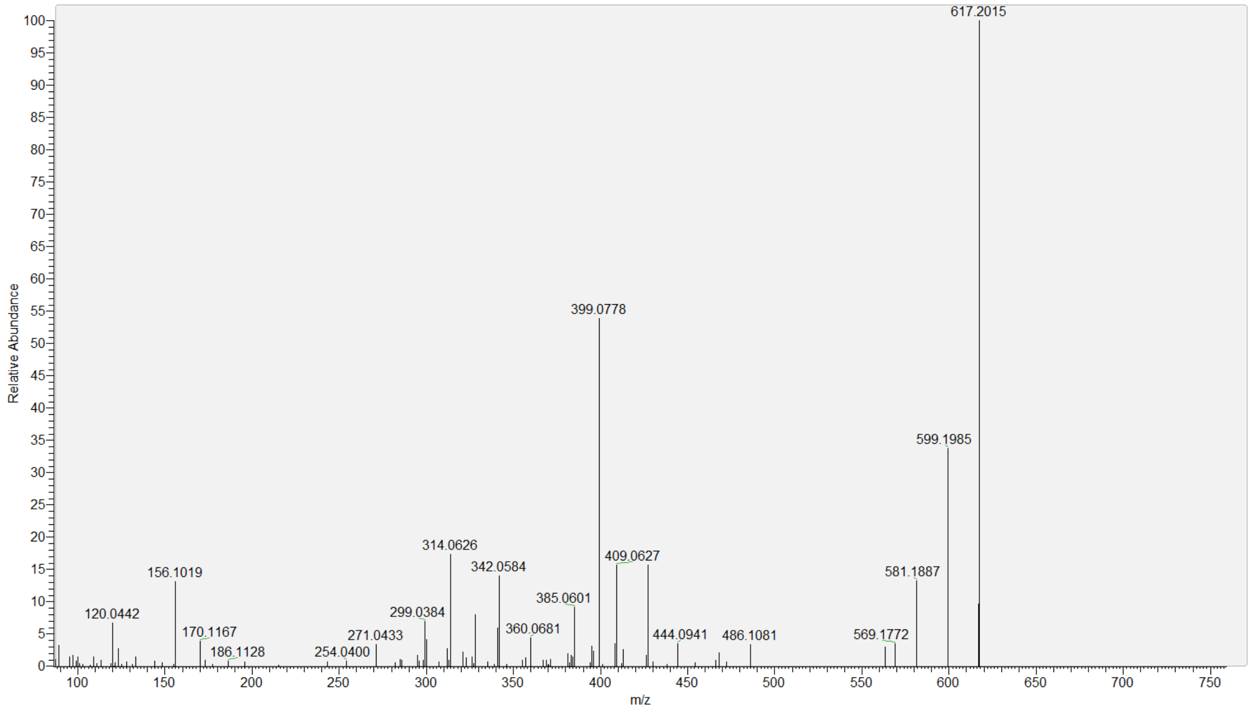

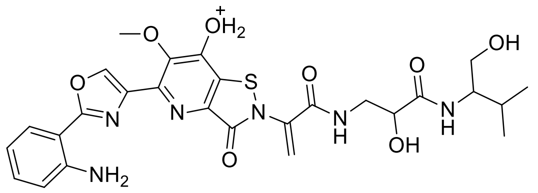

Chemical Formula: $C_{27}H_{31}N_6O_8S^+$
Exact Mass: 599.19186

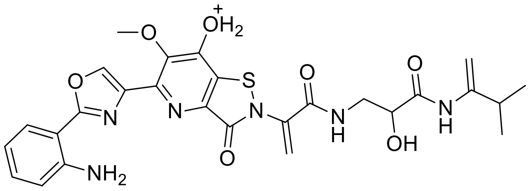

Chemical Formula: $C_{27}H_{29}N_6O_7S^+$
Exact Mass: 581.18129

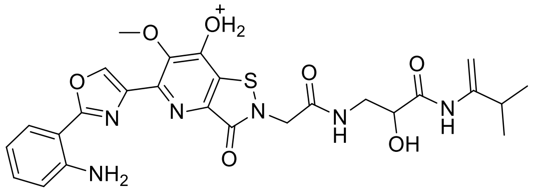

Chemical Formula: $C_{26}H_{29}N_6O_7S^+$
Exact Mass: 569.18129

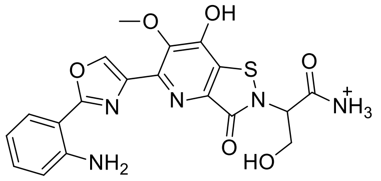

Chemical Formula: $C_{19}H_{18}N_5O_6S^+$
Exact Mass: 444.09723

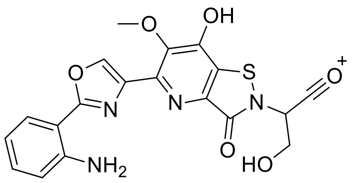

Chemical Formula: $C_{19}H_{15}N_4O_6S^+$
Exact Mass: 427.07068

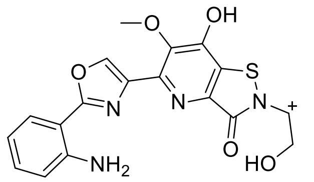

Chemical Formula: $C_{18}H_{15}N_4O_5S^+$
Exact Mass: 399.07577

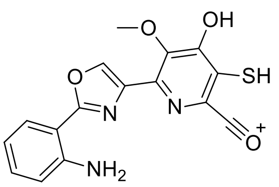

Chemical Formula: $C_{16}H_{12}N_3O_4S^+$
Exact Mass: 342.05430

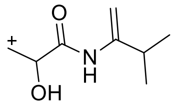

Chemical Formula: $C_8H_{14}NO_2^+$
Exact Mass: 156.10191

Figure S9: Putative Structure of levesquamide C (A). TIC and EIC of levesquamide C (B). MS^1 spectra for levesquamide C (C). MS^2 fragmentation spectra for levesquamide C (D). Predicted observed fragments for levesquamide C (E).

Putative structure for Levesquamide D [M+H] 601.2641 *m/z.* (loss of methoxy)

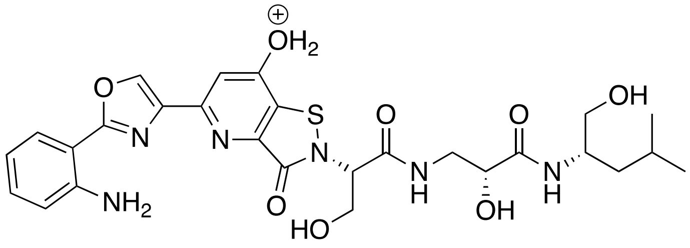

Chemical Formula: $C_{27}H_{33}N_6O_8S^+$
Exact Mass: 601.2075

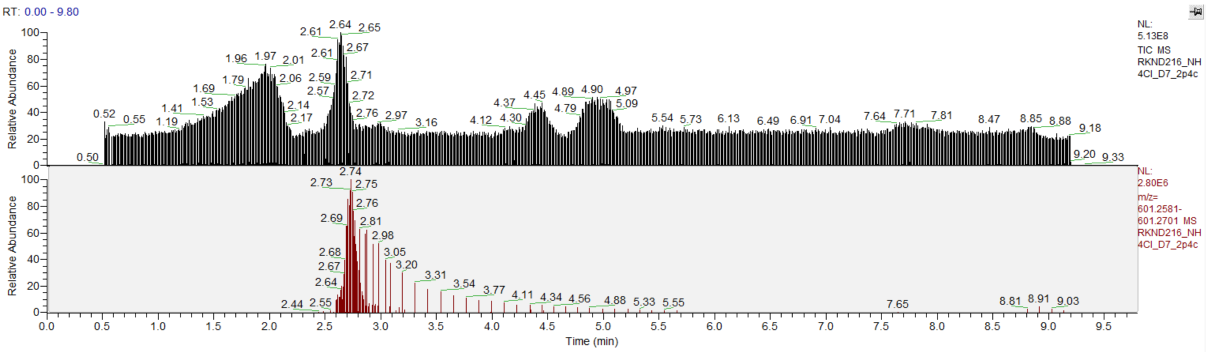

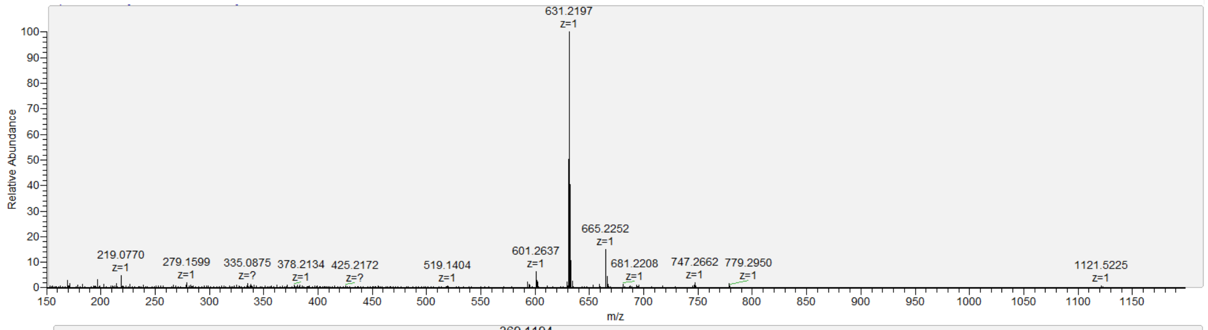

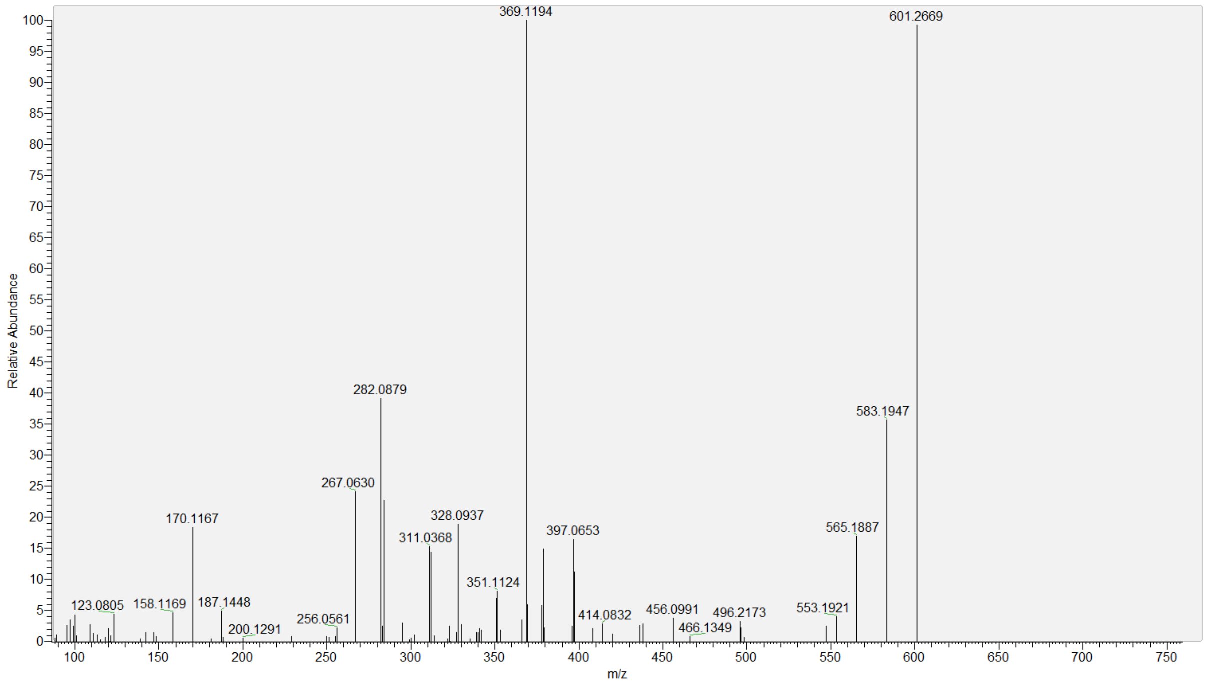

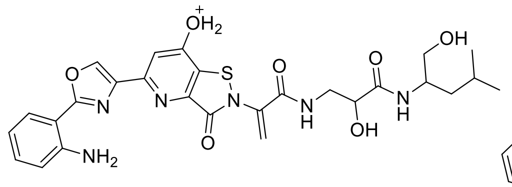

Chemical Formula: $C_{27}H_{31}N_6O_7S^+$
Exact Mass: 583.19694

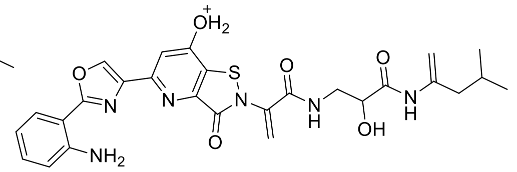

Chemical Formula: $C_{27}H_{29}N_6O_6S^+$
Exact Mass: 565.18638

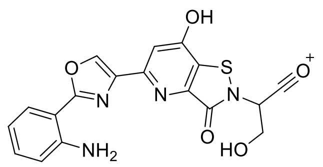

Chemical Formula: $C_{18}H_{13}N_4O_5S^+$
Exact Mass: 397.06012

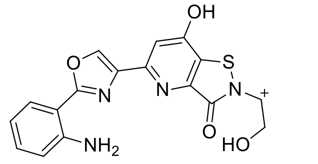

Chemical Formula: $C_{17}H_{13}N_4O_4S^+$
Exact Mass: 369.06520

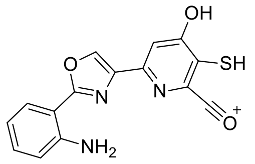

Chemical Formula: $C_{15}H_{10}N_3O_3S^+$
Exact Mass: 312.04374

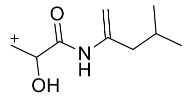

Chemical Formula: $C_9H_{16}NO_2^+$
Exact Mass: 170.11756

Figure S10:Putative structure of levesquamide D (A). TIC and EIC of levesquamide D (B). MS^1 spectra for levesquamide D (C). MS^2 fragmentation spectra for levesquamide D (D). Predicted observed fragments for levesquamide D (E).

Other Levesquamide analogues identified by through GNPS clustering.

Levesquamide E [M+H] 659.2151 *m/z,* $C_{30}H_{39}N_6O_9S^+$.

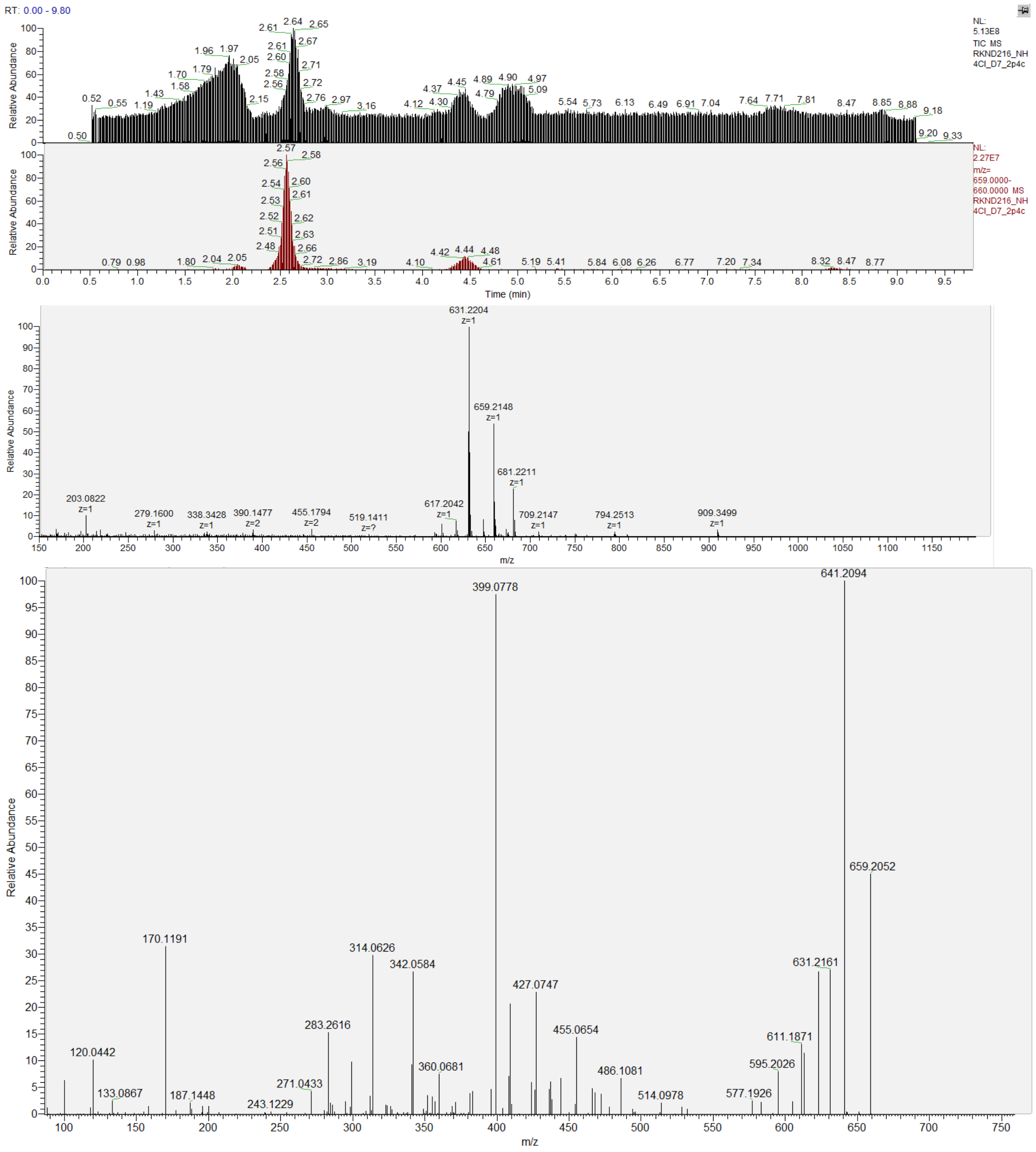

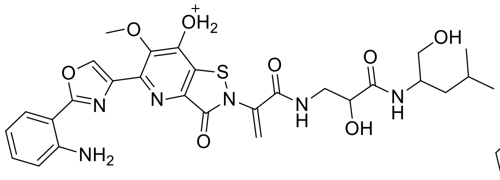

Chemical Formula: $C_{28}H_{33}N_6O_8S^+$
Exact Mass: 613.20751

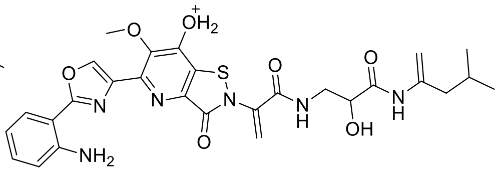

Chemical Formula: $C_{28}H_{31}N_6O_7S^+$
Exact Mass: 595.19694

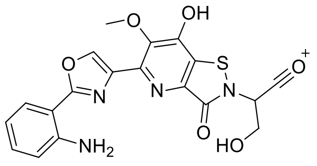

Chemical Formula: $C_{19}H_{15}N_4O_6S^+$
Exact Mass: 427.07068

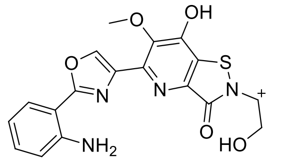

Chemical Formula: $C_{18}H_{15}N_4O_5S^+$
Exact Mass: 399.07577

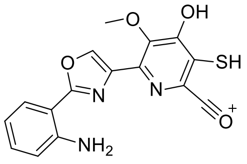

Chemical Formula: $C_{16}H_{12}N_3O_4S^+$
Exact Mass: 342.05430

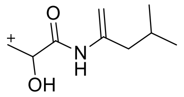

Chemical Formula: $C_9H_{16}NO_2^+$
Exact Mass: 170.11756

Figure S11: TIC and EIC of levesquamide E (A). MS^1 spectra for levesquamide E (B). MS^2 fragmentation spectra for levesquamide E (C). Predicted observed fragments for levesquamide E (D).

Levesquamide F [M+H] 747.2669 *m/z.*

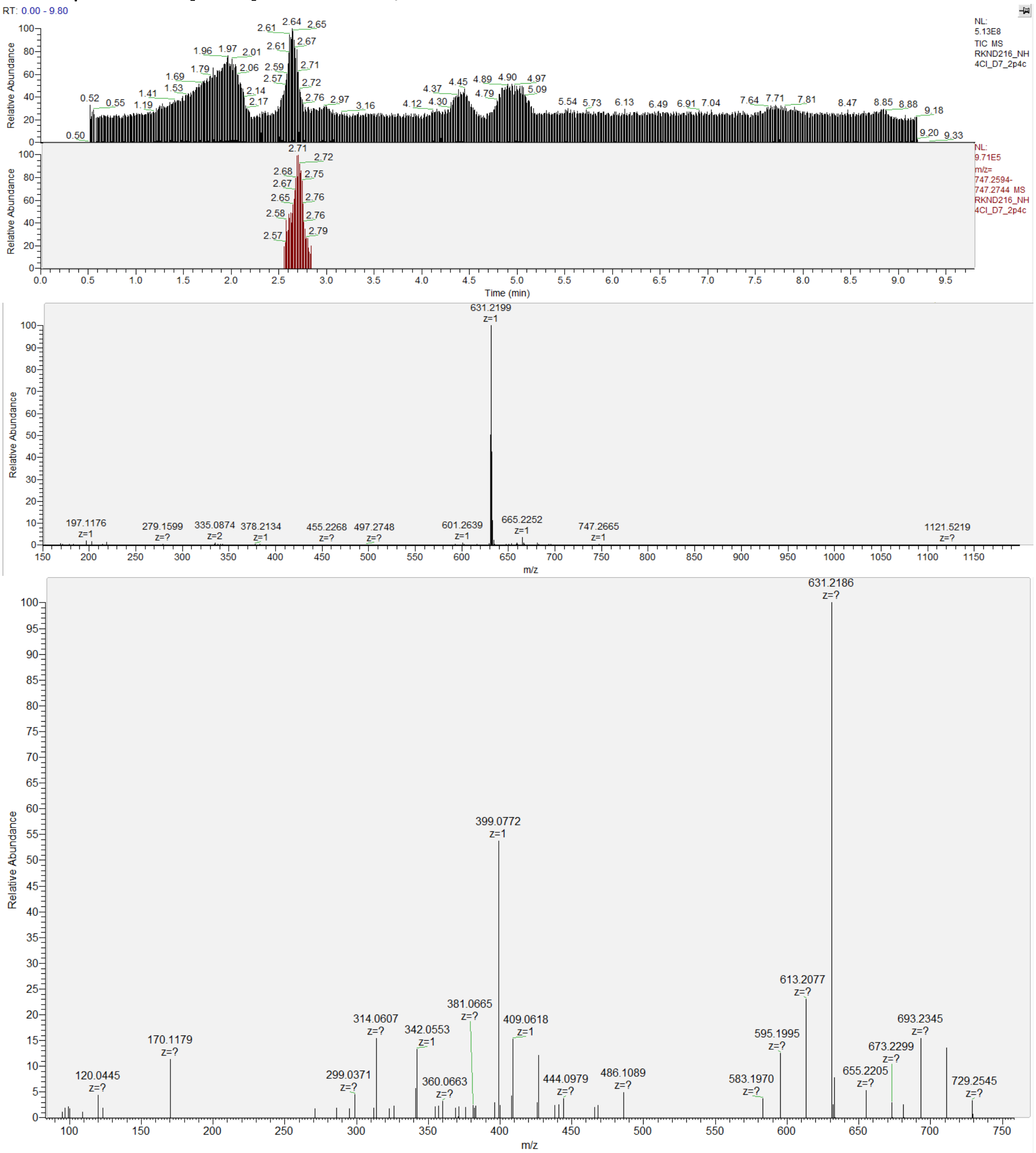

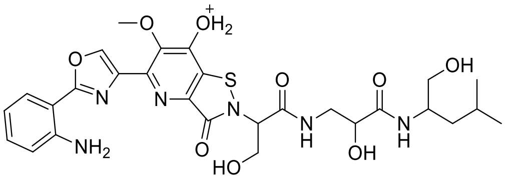

Chemical Formula: $C_{28}H_{35}N_6O_9S^+$
Exact Mass: 631.21807

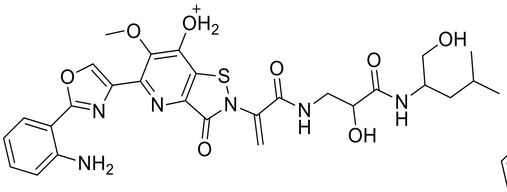

Chemical Formula: $C_{28}H_{33}N_6O_8S^+$
Exact Mass: 613.20751

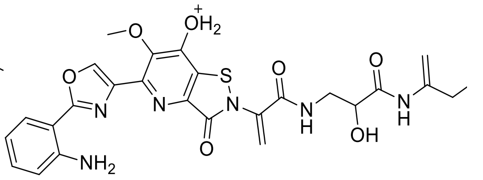

Chemical Formula: $C_{28}H_{31}N_6O_7S^+$
Exact Mass: 595.19694

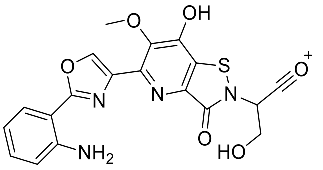

Chemical Formula: $C_{19}H_{15}N_4O_6S^+$
Exact Mass: 427.07068

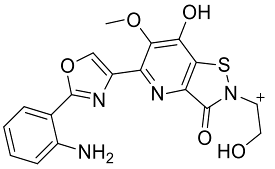

Chemical Formula: $C_{18}H_{15}N_4O_5S^+$
Exact Mass: 399.07577

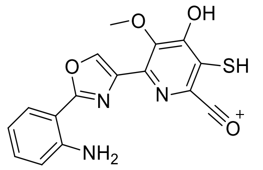

Chemical Formula: $C_{16}H_{12}N_3O_4S^+$
Exact Mass: 342.05430

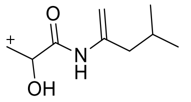

Chemical Formula: $C_9H_{16}NO_2^+$
Exact Mass: 170.11756

Figure S12: TIC and EIC of levesquamide F (A). MS^1 spectra for levesquamide F (B). MS^2 fragmentation spectra for levesquamide F (C). Predicted observed fragments for levesquamide F (D).

Levesquamide G [M+H] 717.2583 *m/z.*

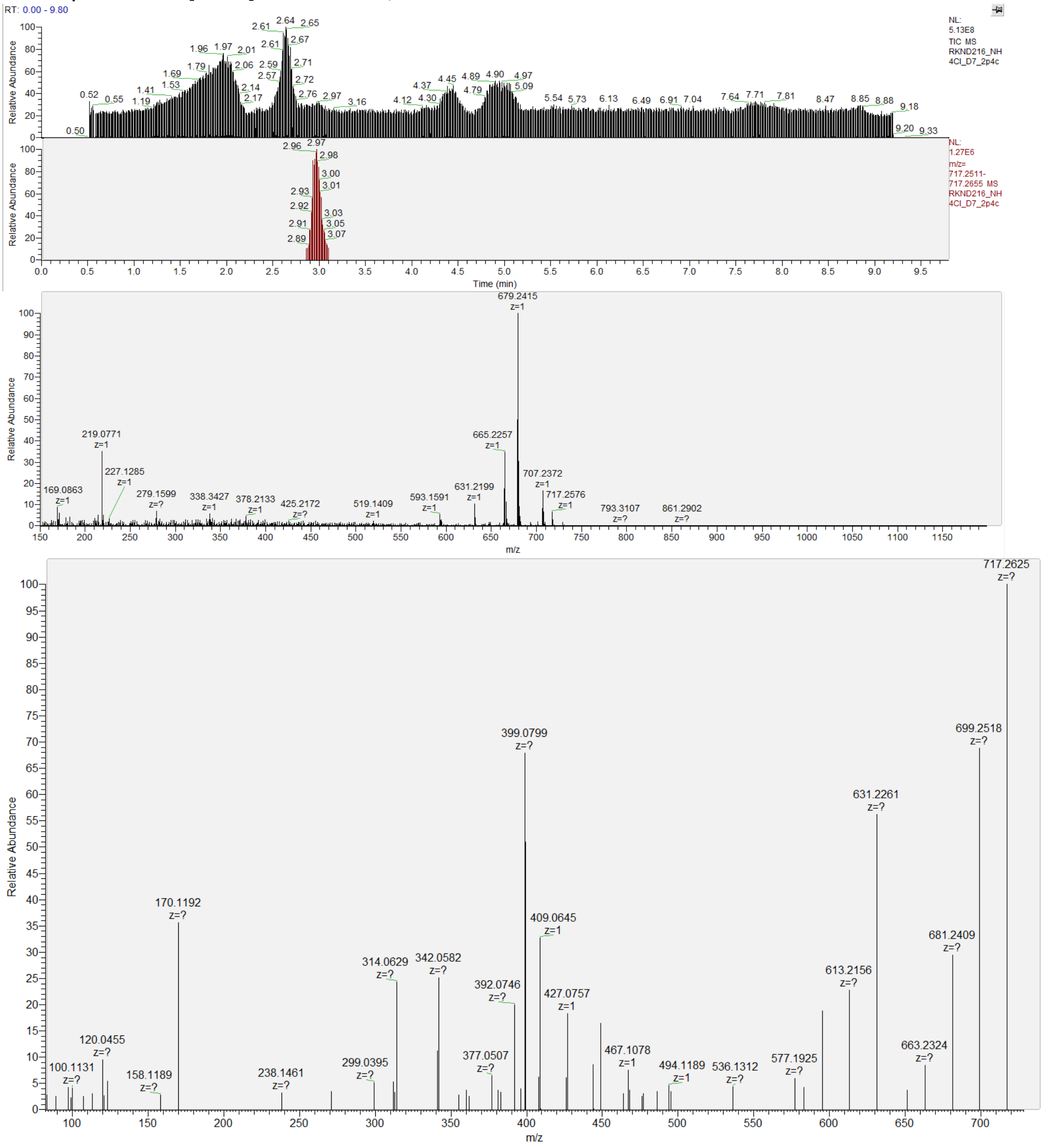

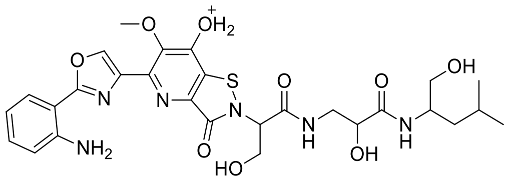

Chemical Formula: $C_{28}H_{35}N_6O_9S^+$
Exact Mass: 631.21807

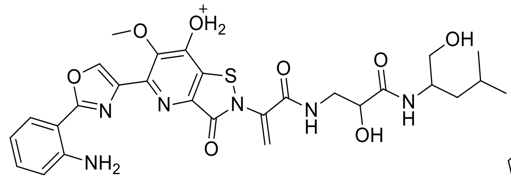

Chemical Formula: $C_{28}H_{33}N_6O_8S^+$
Exact Mass: 613.20751

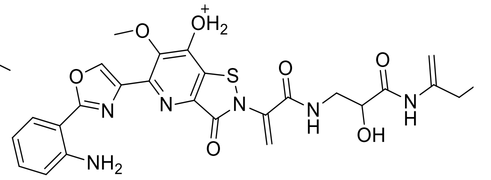

Chemical Formula: $C_{28}H_{31}N_6O_7S^+$
Exact Mass: 595.19694

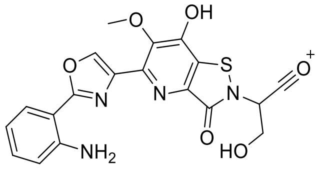

Chemical Formula: $C_{19}H_{15}N_4O_6S^+$
Exact Mass: 427.07068

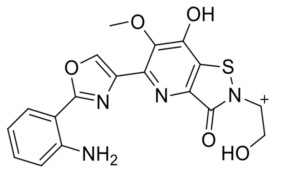

Chemical Formula: $C_{18}H_{15}N_4O_5S^+$
Exact Mass: 399.07577

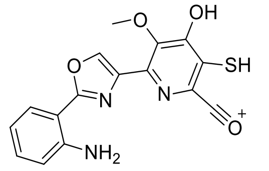

Chemical Formula: $C_{16}H_{12}N_3O_4S^+$
Exact Mass: 342.05430

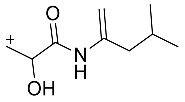

Chemical Formula: $C_9H_{16}NO_2^+$
Exact Mass: 170.11756

Figure S13: TIC and EIC of levesquamide G (A). MS^1 spectra for levesquamide G (B). MS^2 fragmentation spectra for levesquamide G (C). Predicted observed fragments for levesquamide G (D).
